# Mitigating the Poisoning Effect of Formate during CO_2_ Hydrogenation to Methanol over Co-Containing Dual-Atom Oxide Catalysts

**DOI:** 10.1021/jacsau.3c00789

**Published:** 2024-02-02

**Authors:** Nazmul Hasan MD Dostagir, Carlo Robert Tomuschat, Kai Oshiro, Min Gao, Jun-ya Hasegawa, Atsushi Fukuoka, Abhijit Shrotri

**Affiliations:** † Institute for Catalysis, Hokkaido University, Kita 21 Nishi 10, Kita-ku, Sapporo, Hokkaido 001-0021, Japan; ‡ Department of Chemistry, TUM School of Natural Sciences, Technical University of Munich, Lichtenbergstraße 4, 85748 Garching, Germany; § Graduate School of Chemical Sciences and Engineering, Hokkaido University, Kita 13 Nishi 8, Kita-ku, Sapporo, Hokkaido 060-8628, Japan; ∥ Institute for Chemical Reaction Design and Discovery, Hokkaido University, Kita 21 Nishi 10, Kita-ku, Sapporo, Hokkaido 001-0021, Japan; ⊥ Interdisciplinary Research Center for Catalytic Chemistry, National Institute of Advanced Industrial Science and Technology, Central 5, 1-1-1 Higashi, Tsukuba, Ibaraki 305-8565, Japan

**Keywords:** CO_2_ hydrogenation, methanol, selectivity control, formate pathway, doped oxide, in situ DRIFTS

## Abstract

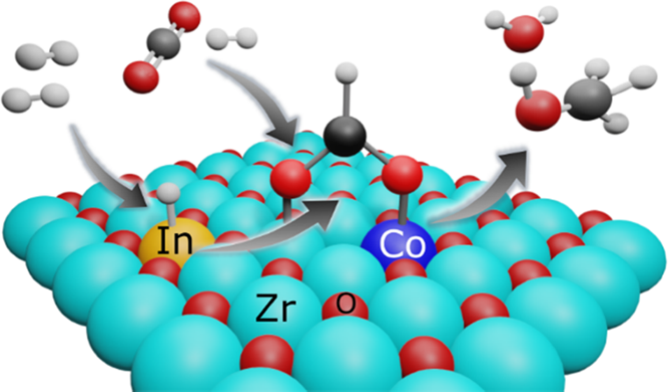

During the hydrogenation of CO_2_ to methanol over mixed-oxide catalysts, the strong adsorption of CO_2_ and formate poses a barrier for H_2_ dissociation, limiting methanol selectivity and productivity. Here we show that by using Co-containing dual-atom oxide catalysts, the poisoning effect can be countered by separating the site for H_2_ dissociation and the adsorption of intermediates. We synthesized a Co- and In-doped ZrO_2_ catalyst (Co–In–ZrO_2_) containing atomically dispersed Co and In species. Catalyst characterization showed that Co and In atoms were atomically dispersed and were in proximity to each other owing to a random distribution. During the CO_2_ hydrogenation reaction, the Co atom was responsible for the adsorption of CO_2_ and formate species, while the nearby In atoms promoted the hydrogenation of adsorbed intermediates. The cooperative effect increased the methanol selectivity to 86% over the dual-atom catalyst, and methanol productivity increased 2-fold in comparison to single-atom catalysts. This cooperative effect was extended to Co–Zn and Co–Ga doped ZrO_2_ catalysts. This work presents a different approach to designing mixed-oxide catalysts for CO_2_ hydrogenation based on the preferential adsorption of substrates and intermediates instead of promoting H_2_ dissociation to mitigate the poisonous effects of substrates and intermediates.

## Introduction

Hydrogenation of CO_2_ to methanol is an effective way to recycle CO_2_,^1−4^ because methanol can be transported in liquid phase and converted to chemicals and fuels.^5^ Achieving high methanol selectivity during CO_2_ hydrogenation is a challenge because of competitive CO formation via the reverse water–gas shift (RWGS) reaction. In the first step of CO_2_ hydrogenation, adsorbed formate species are formed, which are an intermediate for both methanol and CO. While methanol is obtained by hydrogenation of formate, its decomposition produces CO.^6−9^ Therefore, to increase methanol selectivity, it is important to suppress CO formation by promoting the hydrogenation of formate.

Recently, oxide catalysts like In_2_O_3_ and mixed oxides of ZrO_2_^10−16^ have emerged as catalysts for CO_2_ hydrogenation with good methanol selectivity. These catalysts exhibit oxygen vacancies adjacent to a metal atom with hydrogen dissociation ability.^17^ Such interfacial sites strongly adsorb CO_2_ and stabilize the intermediate formate species.^13,18−20^ However, strong adsorption of CO_2_ and formate can poison the active site and limit H_2_ dissociation.^21−26^ For example, catalysts containing atomically dispersed In and Zn on ZrO_2_ cannot dissociate H_2_ efficiently under a CO_2_ environment.^27,28^ As a result, the hydrogenation of formate species stabilized over the catalyst is hindered and their decomposition produces CO.^27−29^

Incorporation of promoters such as metal single atoms and nanoparticles can increase the availability of dissociated H_2_ species.^30−45^ Although the addition of a promoter increases the methanol yield, it simultaneously decreases methanol selectivity because improved hydrogen dissociation also promotes the competitive RWGS reaction.^32,36−39,41,42,46−48^ Adding metal promoters for hydrogen dissociation creates independent active sites that catalyze the RWGS reaction and total reduction of CO_2_ to methane.^49,50^

Here we introduce an alternative approach for mitigating the poisonous effect by creating a site for the adsorption of CO_2_ and stabilization of formate by introducing a Co single atom into the ZrO_2_ structure. This preferential adsorption of CO_2_ at a site different from the metal center for H_2_ dissociation reduces the poisoning effect and improves both methanol selectivity and productivity by suppressing the RWGS reaction. We show that in a dual-atom oxide catalyst containing Co- and In-doped ZrO_2_ (Co–In–ZrO_2_), the Co–Zr interfacial site adsorbs CO_2_ and In promotes its hydrogenation. Over the Co–In–ZrO_2_ catalyst, methanol selectivity is less dependent on the H_2_ and CO_2_ partial pressure because the poisoning effect of adsorbed species is mitigated as observed by change in the order of the reaction with respect to CO_2_ and H_2_. The concept of introducing Co single atoms to mitigate the poisonous effect was also extended to Co–Zn–ZrO_2_ and Co–Ga–ZrO_2_ dual-atom systems, demonstrating the robustness of this approach. In the Co-based catalysts reported so far for methanol production (for example: Co_3_O_4_–MnO_*x*_ and In–Co core–shell systems), Co promotes the H_2_ dissociation.^34,35,51,52^ The better H_2_ dissociation ability improves the hydrogenation of the formate intermediate to methanol. Although these Co-based systems improved methanol productivity, methanol selectivity decreased due to methane and CO formation over the metallic and oxidic Co surface. This study provides a different approach to catalyst design for CO_2_ hydrogenation that prioritizes interfacial sites for facilitating the adsorption of CO_2_ and stabilization of formate and complementing them with elements that dissociate H_2_.

## Experimental Section

### Materials

All chemicals were used without further purification, unless otherwise noted. Aqueous ammonia solution (29–30 wt % NH_3_), formic acid, indium(III) nitrate trihydrate (In(NO_3_)_3_·3H_2_O), cobalt(II) nitrate hexahydrate (Co(NO_3_)_2_·6H_2_O), zirconyl nitrate dihydrate (ZrO(NO_3_)_2_·2H_2_O), zinc(II) nitrate hexahydrate (Zn(NO_3_)_2_·6H_2_O), and gallium(III) nitrate *n*-hydrate (Ga(NO_3_)_3_·*n*H_2_O) were purchased from FUJIFILM Wako Pure Chemical Corporation.

### Catalyst Preparation

Doped ZrO_2_ catalysts were prepared by using the coprecipitation method. In a typical method to synthesize the Co–In–ZrO_2_ catalyst, Co(NO_3_)_2_·6H_2_O (1 mmol), In(NO_3_)_3_·3H_2_O (0.5 mmol), and ZrO(NO_3_)_2_·2H_2_O (8.5 mmol) were dissolved in a round-bottomed flask containing 150 mL of deionized water. Diluted aqueous NH_4_OH (9 wt % NH_3_) solution was added dropwise to the metal solution under vigorous stirring until the pH became 9. The formed precipitate was aged for 1 h under stirring at room temperature. The precipitate was recovered by centrifugation and washed with deionized water until the pH of the supernatant became neutral. The solid was dried at 130 °C for 12 h. The final catalyst was obtained after calcination in a muffle furnace at 500 °C for 3 h (ramp rate of 2 °C min^–1^) under static air. Dual-atom oxide catalysts of Zn and Ga were prepared using the same method, and the catalysts were named Co-M-ZrO_2_ (M = In/Zn/Ga). Single-atom oxides of Co-doped ZrO_2_ (Co–ZrO_2_), *M*-doped ZrO_2_ (M-ZrO_2_), and undoped ZrO_2_ were prepared using the same procedure. Metal loadings were calculated in atom % of the respective metal in relation to the total metal content. The loadings of Co and M (In, Zn, Ga) were kept constant at 10 and 5 atom %, respectively.

For example, the loading of Co (*L*_Co_) in atom % was calculated as
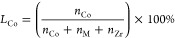
1where *n*_Co_, *n*_M_, and *n*_Zr_ are the mol of Co, M, and Zr, respectively.

### Catalyst Characterization

Catalysts were characterized using X-ray diffraction (XRD), N_2_ adsorption–desorption experiment, X-ray photoelectron spectroscopy (XPS), scanning transmission electron microscopy (STEM), H_2_ temperature-programmed reduction, CO_2_ temperature-programmed desorption, temperature-programmed formic acid decomposition, and diffuse reflectance infrared Fourier transform spectroscopy (DRIFTS) techniques. See Supporting Information for detailed procedures.

### Catalytic Testing Procedure

Catalytic activity for CO_2_ hydrogenation was evaluated in a custom-built stainless-steel fixed-bed flow reactor system (Figure S9). Typically, 200 mg of catalyst was loaded into the reactor and held in place with quartz wool. A thermocouple was inserted into the reactor to measure the catalyst bed temperature. H_2_ and CO_2_ were supplied by two mass-flow controllers (Bronkhorst Japan K.K.) and mixed before the inlet of the reactor. The reactor was pressurized using a mixture of H_2_ and CO_2_ having the ratio H_2_/CO_2_ = 4:1 via a backpressure regulator (Swagelok Japan FST Co., Ltd.). After the system pressure was stable, the reactor temperature was increased to the desired value. The total flow rate was 100 mL min^–1^ to maintain the gas-hourly space velocity at 30,000 mL h^–1^ g_cat_^–1^. Reaction products and unreacted CO_2_ were quantified online by a Shimadzu GC 8A gas chromatograph (Shimdazu Corp.) equipped with two channels and a thermal conductivity detector. Porapak Q and Molsieve 5 Å packed columns were used for the separation of CO_2_, MeOH, and CO, respectively. The gas line from the outlet of the reactor to the inlet of the GC was heated to 170 °C to prevent condensation of the liquid products. See Supporting Information for the mathematical equations for calculating CO_2_ conversion, selectivity of products, and space-time yield of CO and MeOH.

## Results and Discussion

### Preparation and Structural Characterization of Oxide Catalysts

All of the doped oxides were prepared by coprecipitation of metal nitrates with ammonia solution followed by washing, drying, and calcination at 500 °C under air. The loading of Co and In in doped catalysts was 10 and 5 atom %, respectively. The surface concentration of Co for Co–In–ZrO_2_ and Co–ZrO_2_ was similar to the theoretical value at 11 atom % as measured by X-ray photoelectron spectroscopy (XPS) (Table S1). The surface concentration of In in Co–In–ZrO_2_ was 11 atom %, roughly two times the theoretical loading. The surface concentration of In in In–ZrO_2_ was 12 atom %, suggesting that In prefers to migrate toward the surface when doped in ZrO_2_. Consequently, the ratio of Co and In on the surface of Co–In–ZrO_2_ was approximately 1:1.

**Figure 1:**
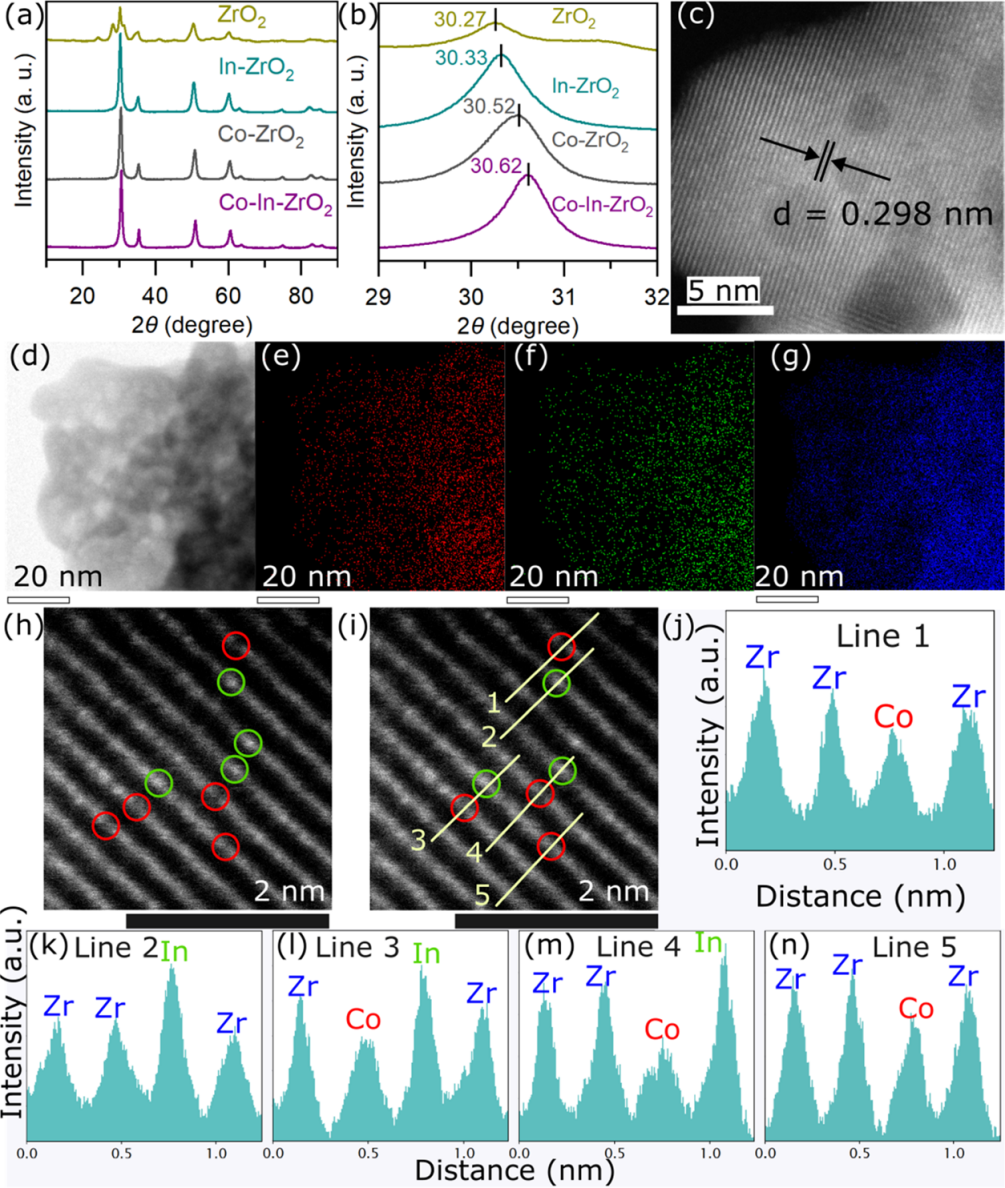
(a) XRD patterns of doped oxides along with (b) the shift of the (101) reflection of *t*-ZrO_2_. (c) HAADF-STEM analysis of Co–In–ZrO_2_ showing the (101) plane of *t*-ZrO_2_ and the corresponding *d* spacing. (d) HAADF-STEM image of the Co–In–ZrO_2_ catalyst with the corresponding elemental mapping of (e) Co (red), (f) In (green), and (g) Zr (blue). (h) Double-aberration-corrected HAADF-STEM image of Co–In–ZrO_2_ showing Co (red circle) and In (green circle) single atoms. (j, n) Line intensity profiles of the selected lines shown in (i).

Doping did not introduce any morphological changes and catalysts showed similar N_2_ adsorption isotherms and surface areas in the range of 65–73 m^2^ g^–1^ (Figure S1 and Table S1). In the X-ray diffraction (XRD) analysis (Figure 1a), diffraction peaks attributed to *t*-ZrO_2_ were observed and peaks for oxides of dopants such as CoO, Co_3_O_4_, and In_2_O_3_ were not apparent. The 2θ value corresponding to the (101) plane of *t*-ZrO_2_ shifted to a higher value (Figure 1b) because of a decrease in interplanar distance in *t*-ZrO_2_ owing to the smaller ionic radii of the dopants (*r*_Co^2+^_ = 0.74 Å, *r*_In^3+^_ = 0.8 Å) as compared to Zr^4+^ (*r*_Zr^4+^_ = 0.84 Å).^53^ The lattice parameter of the *t*-ZrO_2_ was reduced with respect to the ionic radii and concentration of the dopant (Figure S2). These results indicate homogeneous doping of metals in the ZrO_2_ matrix without the formation of oxides of In and Co.

In the high-angle annular dark-field scanning transmission electron microscopy (HAADF-STEM) image of Co–In–ZrO_2_ only the *t*-ZrO_2_ phase was visible (Figure 1c). Elemental mapping using energy dispersive X-ray (EDX) analysis (Figure 1d–g) revealed a homogeneous distribution of Co and In atoms. HAADF-STEM image and elemental mapping of Co–ZrO_2_ (Figure S3a–c) and In-ZrO_2_ (Figure S3d–f) also showed a homogeneous distribution of Co and In atoms, respectively. The individual In and Co atoms in Co–In–ZrO_2_ were identified by double-aberration-corrected HAADF-STEM analysis. The line intensity profile showed areas of higher and lower intensity for the presence of In and Co atoms (Figure 1h–n), respectively, according to the Z-contrast, relative to their atomic number. These results show that In and Co atoms replaced Zr atoms in the crystal structure and dopant atoms were observed in proximity of each other by way of random distribution within the ZrO_2_ matrix.

### Catalytic Performance of Oxide Catalysts

**Figure 2:**
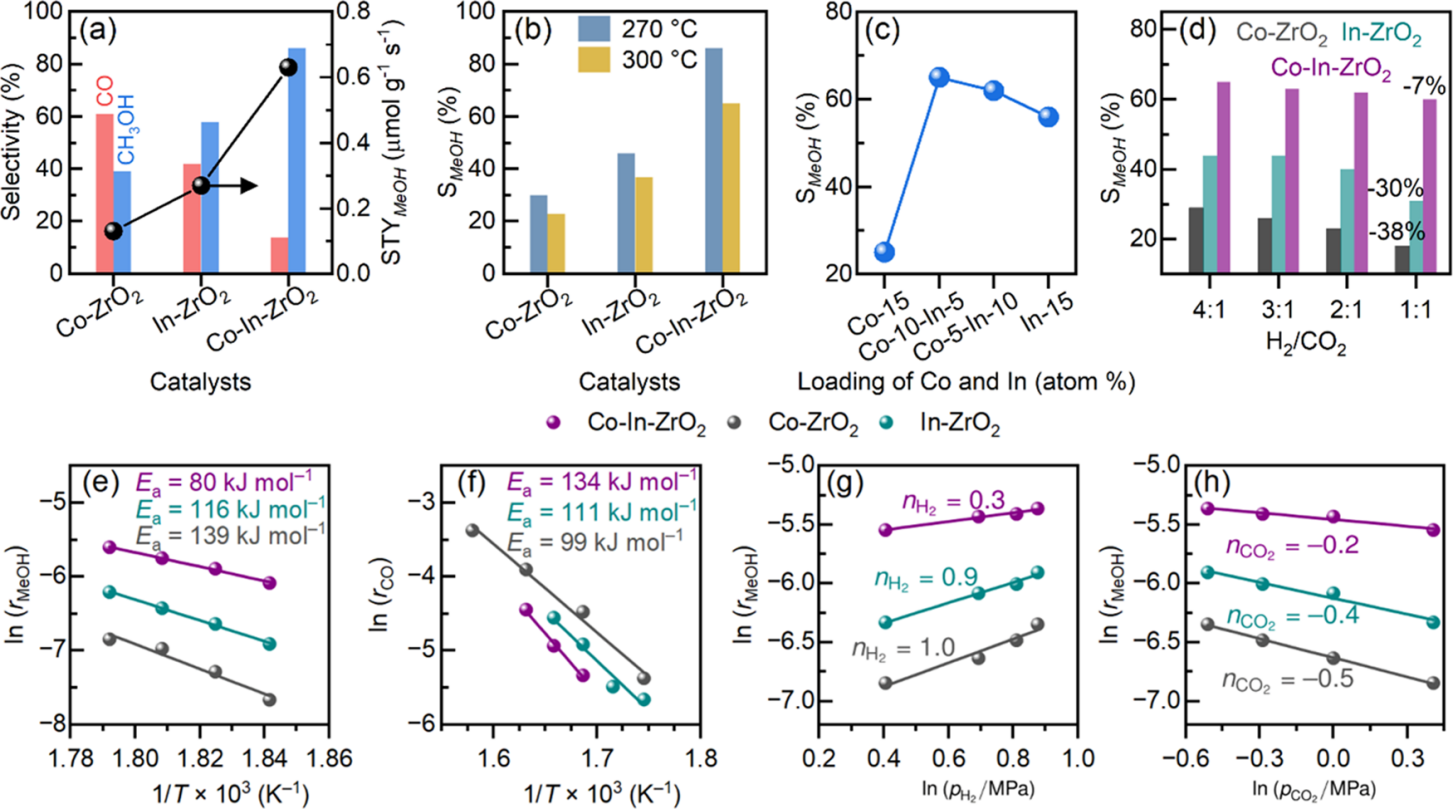
(a) Comparison of the catalytic activities of Co–ZrO_2_, In–ZrO_2_, and Co–In–ZrO_2_. Reaction conditions: 270 °C, 3 MPa, 30,000 mL h^–1^ g_cat_^–1^, H_2_/CO_2_ = 4. (b) Comparison of methanol selectivity at 300 and 270 °C over different catalysts at the same CO_2_ conversion. CO_2_ conversion was maintained at 1 and 2.7% for all catalysts at 270 and 300 °C, respectively. The same CO_2_ conversion was achieved by changing the space velocity. (c) Selectivity of methanol over doped ZrO_2_ catalysts with a total dopant loading of 15% at the same CO_2_ conversion level of 2.7%. Reaction conditions: 300 °C, 3 MPa, SV = 30,000–45,000 mL h^–1^ g_cat_^–1^, H_2_/CO_2_ = 4. (d) Methanol selectivity under varying H_2_/CO_2_ ratio for Co–ZrO_2_, In–ZrO_2_, and Co–In–ZrO_2_. The percentages denote a relative decrease in methanol selectivity. Reaction conditions: 300 °C, 3 MPa, 30,000 mL h^–1^ g_cat_^–1^. Arrhenius plot for the calculation of the apparent activation energy of (e) methanol formation and (f) CO formation over doped oxide catalysts. Determination of the order of methanol formation with respect to (g) H_2_ and (h) CO_2_. The rate of products was taken in mol g_cat_^–1^ h^–1^ units for calculations.

The performance of all catalysts for catalytic CO_2_ hydrogenation was evaluated in a stainless-steel fixed-bed flow reactor (Figure S4). Undoped ZrO_2_ showed negligible CO_2_ conversion (Table S2). While the CO_2_ conversion over the Co–In–ZrO_2_ catalyst was slightly higher than that over binary oxide catalysts, the methanol selectivity was very different (Figure 2a and Table S2). At 270 °C the methanol selectivity over Co–In–ZrO_2_ was 86%, whereas the methanol selectivity over In–ZrO_2_ and Co–ZrO_2_ was 58 and 39%, respectively. High methanol selectivity was achieved at the same CO_2_ conversion level, further confirming the promotional effect of Co–In–ZrO_2_ (Figure 2b). The difference in selectivity between dual- and single-atom-doped catalysts was maintained at 300 °C (Figure 2b and Table S2). The space-time yield (STY) of methanol over Co–In–ZrO_2_ at 300 °C was 1.3 μmol g^–1^ s^–1^, which was higher than the combined methanol STY of the binary oxides (Table S2). The selectivity and STY of CO were lower over Co–In–ZrO_2_ in comparison to those over binary oxides (Table S2 and Figure S5). These results suggest that the methanol formation was favored over the Co–In–ZrO_2_ catalyst, and the RWGS reaction was suppressed. Figure 2c shows the change in methanol selectivity at the same conversion level with respect to change in composition of Co and In, while keeping the total dopant loading constant at 15 atom %. Methanol selectivity was independent of total cation loading, and the highest methanol selectivity was obtained at an optimum loading of Co 10 atom % and In 5 atom %. It is evident that the presence of Co and In atoms in the vicinity of the ZrO_2_ surface promoted methanol formation. The spent Co–In–ZrO_2_ catalyst was analyzed using XRD and EDX analysis and no change in the catalyst structure was observed (Figure S6). For comparison, we also prepared a Co/In–ZrO_2_ catalyst having metallic Co nanoparticles, which showed a high selectivity for methane during CO_2_ hydrogenation (Figure S7), emphasizing the role of Co single atoms in avoiding the formation of side products.

Methanol formation in CO_2_ hydrogenation is associated with the hydrogen utilization ability of the catalyst.^54^ Changing the H_2_/CO_2_ ratio from 4:1 to 1:1 over the Co–In–ZrO_2_ catalyst, the methanol selectivity decreased by only 7% of the original value (Figure 2d). In comparison, over Co–ZrO_2_ and In–ZrO_2_, the methanol selectivity decreased by 38 and 30%, respectively. Therefore, the dual-atom oxide catalyst was more effective in terms of utilization of hydrogen for methanol production under hydrogen-lean conditions.

To better understand the ability of Co–In–ZrO_2_ to produce better methanol under low H_2_ partial pressure, we calculated the order of the methanol formation reaction with respect to the reactants and the apparent activation energy (*E*_a_) for the formation of methanol and CO. The *E*_a_ for methanol formation (Figure 2e) was the highest for Co–ZrO_2_ (139 kJ mol^–1^) followed by In–ZrO_2_ (116 kJ mol^–1^) and the lowest for the Co–In–ZrO_2_ oxide (80 kJ mol^–1^). The *E*_a_ for the formation of CO followed the reverse trend (Figure 2f). For Co–ZrO_2_, the order of methanol formation with respect to H_2_ (*n*_H_2__) was 1.0 and that with respect to CO_2_ (*n*_CO_2__) was −0.5 (Figure 2g,h). The values of *n*_H_2__ and *n*_CO_2__ for In–ZrO_2_ were similar at 0.9 and −0.4, respectively. Therefore, over single-atom doped oxide catalysts the activation of H_2_ was the limiting process and a higher partial pressure of H_2_ was needed for methanol formation. The *n*_CO_2__ values of −0.4 and −0.5 for single-atom doped oxides indicate that CO_2_ adsorption over the surface inhibited methanol formation. These results are in line with the reported literature^55^ and it has been shown that methanol selectivity reduces over oxide catalysts because the adsorption of intermediates and H_2_ dissociation occur on the same site.^27,28^ In comparison, over the Co–In–ZrO_2_ catalyst the *n*_H_2__ and *n*_CO_2__were 0.3 and −0.2, respectively. The higher *n*_CO_2__ was due to the mitigation of the adverse effect of poisoning due to the adsorbed intermediates. Also, the much lower value of *n*_H_2__ indicates that H_2_ activation over the dual-atom oxide catalyst was more facile than that over single-atom catalysts. We propose that over the Co–In–ZrO_2_ catalyst, the H_2_ activation and CO_2_ adsorption occur on different yet proximal sites, and methanol formation is promoted because of the cooperative effect between Co and In.

### Site for CO_2_ Adsorption in the Dual-Atom Oxide Catalyst

**Figure 3:**
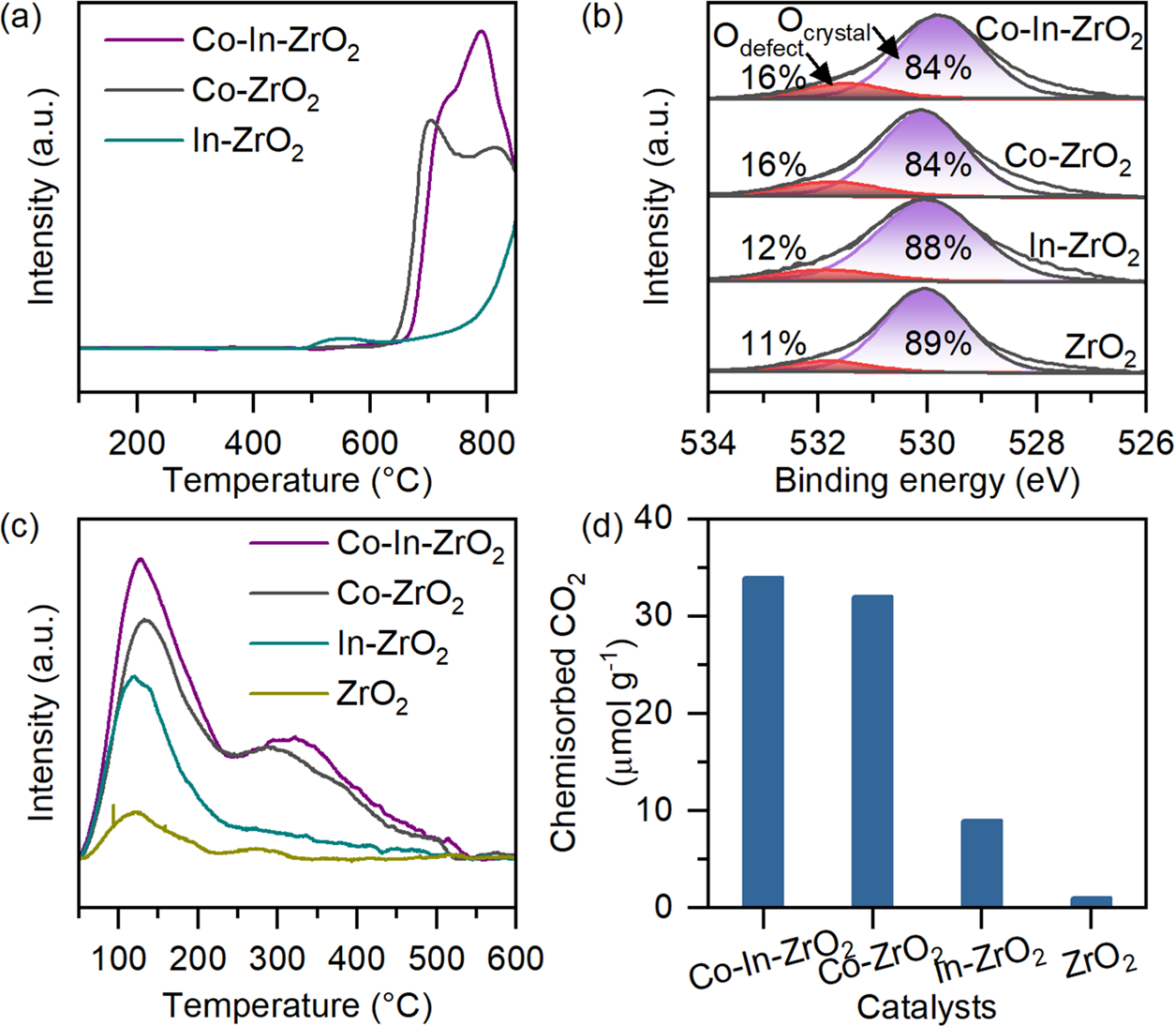
(a) H_2_ TPR of doped catalysts. (b) O 1s XPS spectra, (c) CO_2_ TPD, and (d) chemisorbed CO_2_ amount of all doped oxides and undoped ZrO_2_.

For all catalysts, in the H_2_ temperature-programmed reduction (H_2_-TPR), no characteristic reduction peaks were observed until 500 °C, indicating that the dopants did not reduce under the reaction condition (Figure 3a). The In and Co dopants maintained their ionic states (In^3+^ and Co^2+^, respectively) as observed in the XPS analysis of the catalysts after the reaction (Figures S8 and S9). We have previously shown that inherent oxygen vacancies are formed in Co–ZrO_2_ due to the charge imbalance and difference in coordination environments between Co^2+^ and Zr^4+^.^56^ The density of defective oxygen species, measured by O 1s XPS of the fresh catalyst (the peak around 531.6 eV), is indicative of the abundance of inherent oxygen vacancy. The relative abundance of defective oxygen species in Co–In–ZrO_2_ (16%) was higher than that in In–ZrO_2_ (12%) and ZrO_2_ (11%), but the same as that in Co–ZrO_2_ (16%) (Figure 3b). However, determination of defective oxygen species by XPS is influenced by the presence of surface −OH species. Because of this reason, estimation of change in the abundance of oxygen vacancy on the surface solely based on O 1s XPS can be ambiguous. To confirm the change in abundance of −OH and oxygen vacancy after Co and In doping on the surface, we checked the relative intensities of the bicarbonate species (HCO_3_*) and carbonate (CO_3_*) species in IR analysis after adsorbing CO_2_ (Figure S10). The formation of HCO_3_* is indicative of the presence of −OH species and increase in CO_3_* species is indicative of the increase in oxygen vacancy on the surface (see Figure S10 and the following discussion). For doped oxides, carbonate formation increased compared to that of pure ZrO_2_ (Figure 10a). For Co–In–ZrO_2_ and Co-ZrO_2_, the carbonate peak intensities were similar and the highest among all of the catalysts. This is in line with the oxygen vacancy abundance indicated by the O 1s XPS analysis. As compared to pure ZrO_2_, addition of dopant decreased the relative abundance of surface −OH species and it decreased the most when Co was present (Figure S10b). Therefore, it can be concluded that doping of Co and In increased the oxygen vacancy on the surface rather than increasing the number of −OH groups.

Oxygen-vacant sites are oxophilic in nature and enhance the adsorption of strongly bonded CO_2_.^17^ In the CO_2_ temperature-programmed desorption (CO_2_-TPD) analysis, two desorption features were observed for all of the catalysts (Figure 3c). The low-temperature feature (100–300 °C) was due to the desorption of physisorbed and weakly adsorbed CO_2_, while the feature around 320 °C was assigned to the desorption of chemisorbed CO_2_. For Co–In–ZrO_2_ both physisorbed and chemisorbed CO_2_ were present in higher amounts. Moreover, the TPD profile of Co–In–ZrO_2_ was similar to that of Co-ZrO_2_, especially in the chemisorbed CO_2_ region (in line with the similar CO_3_* peak intensity in the IR analysis of adsorbed CO_2_ shown in Figure S10). Comparing the amounts of chemisorbed CO_2_ for all catalysts, it was evident that CO_2_ chemisorption in Co–In–ZrO_2_ was related to the doping of Co (Figure 3d). Therefore, Co sites are responsible for the CO_2_ chemisorption in Co–In–ZrO_2_.

### Stabilization of Formate on the Dual-Atom Oxide Catalyst

**Figure 4:**
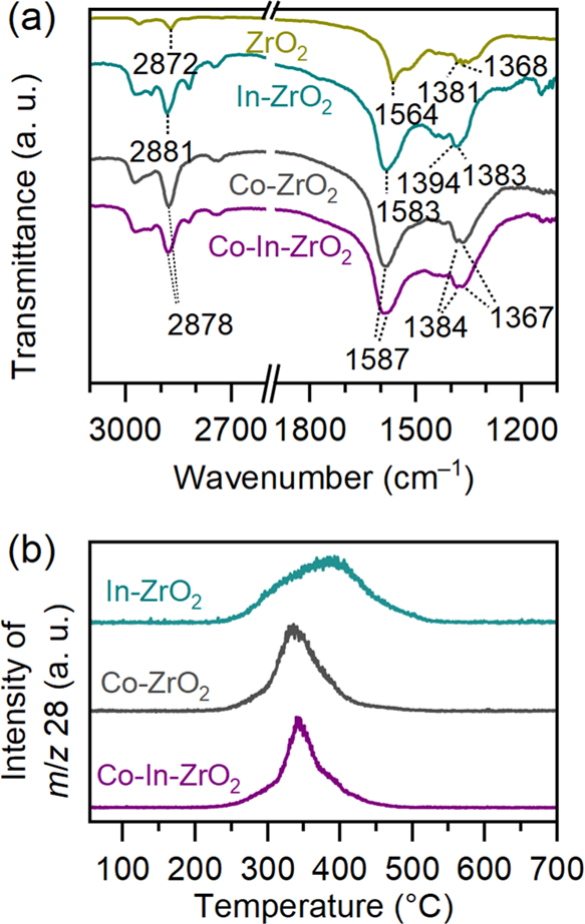
(a) Comparison of formate peak positions during the in situ DRIFTS experiment. Reaction conditions for DRIFTS experiment: 300 °C, 0.1 MPa, and H_2_/CO_2_ = 4:1. For undoped ZrO_2_, the reaction temperature was 340 °C. (b) Mass profile of CO during formic acid decomposition to CO over doped oxide catalysts.

In CO_2_ hydrogenation, formate is formed by hydrogenation of adsorbed CO_2_ and it is the key intermediate for methanol formation over oxide catalysts.^11,57^ In order to observe the nature of formate species over different catalysts, in situ diffuse reflectance infrared Fourier transform spectroscopy (DRIFTS) (Figure S11) analysis was performed at 300 °C, 0.1 MPa, and H_2_/CO_2_ = 4:1. Figure 4a shows the comparison of formate peak positions for all catalysts after 100 min of reaction. In comparison to undoped ZrO_2_, the positions of the formate peaks were shifted in the presence of doped oxides. Bidentate formate bonded with two Zr atoms is the most stable formate configuration in undoped ZrO_2_.^14,58,59^ In the doped oxides, stabilization of formate at the interfacial M-Zr (M = Co or In) site causes a shift in peak position.^28,56,60^ It is evident that the nature of formate species over Co–In–ZrO_2_ and Co–ZrO_2_ was the same, owing to the similar peak position in the DRIFTS analysis. Therefore, formate species in the dual-atom catalyst were present on the Co–Zr interfacial site.

Further confirmation of the tendency of formate species to preferentially adsorb on Co sites was obtained by temperature-programmed decomposition of adsorbed formic acid over the catalyst surface (Figure 4b). The decomposition of adsorbed formic acid over Co–In–ZrO_2_ resulted in CO formation with a peak top at 340 °C. The decomposition profile was similar to Co–ZrO_2_ but different from In–ZrO_2_, which exhibited a broad peak centered at 390 °C. These results confirm that the oxygen-vacant site created by doping of Co in Co–In–ZrO_2_ was responsible for CO_2_ adsorption and stabilization of formate after the hydrogenation of CO_2_.

### Cooperation between Co and In Atoms for Methanol Formation

**Figure 5:**
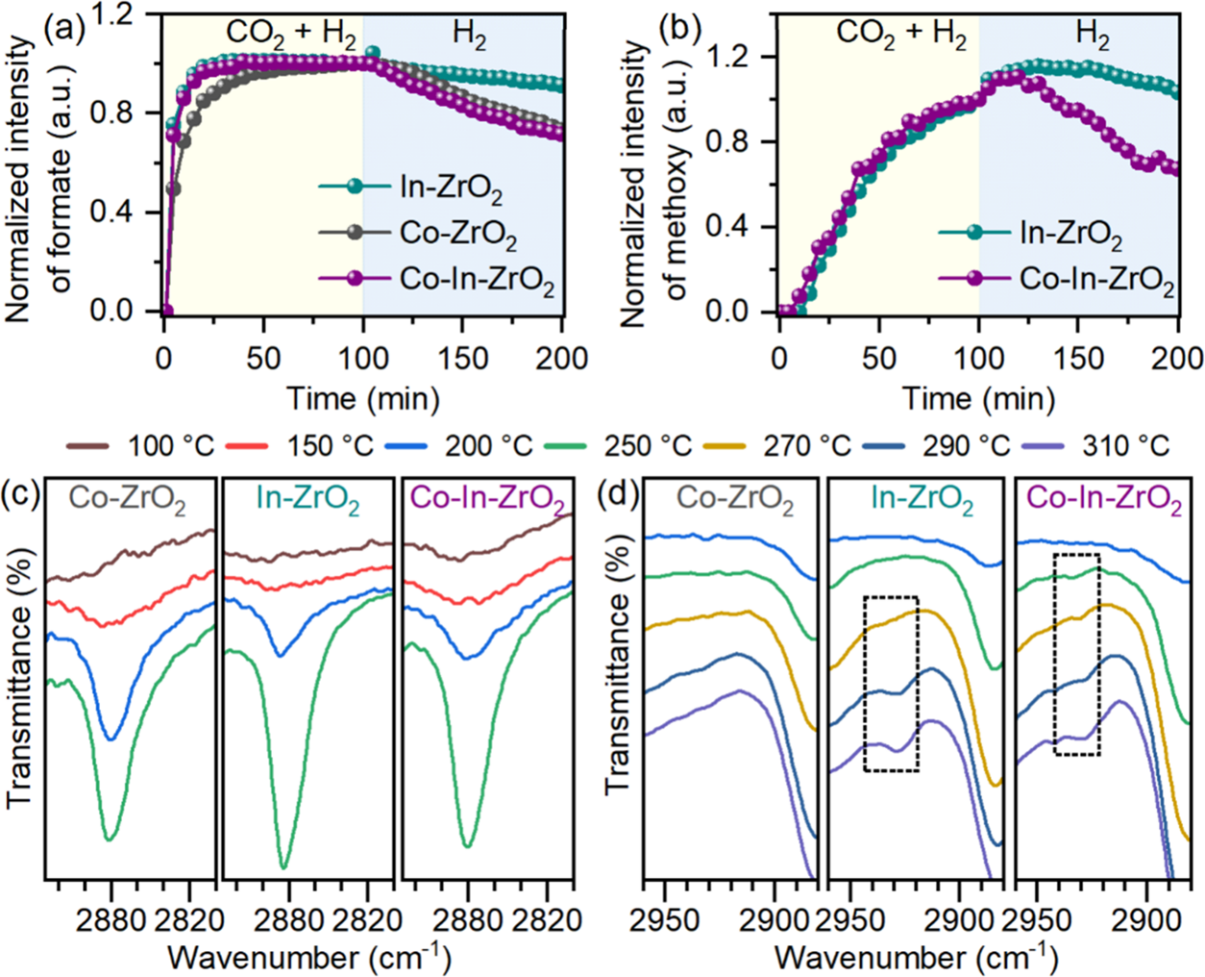
(a, b) Normalized intensity of formate and methoxy species during in situ DRIFTS experiment over Co–ZrO_2_, In–ZrO_2_, and Co–In–ZrO_2_, based on the DRIFTS spectra shown in Figures S10–S12. For each catalyst, the peak intensity was normalized using the peak intensity of the respective species at 100 min during the reaction. Reaction condition: 300 °C, 0.1 MPa, H_2_/CO_2_ = 4:1. (c, d) Temperature-dependent evolution of formate and methanol over different catalysts during in situ DRIFTS analysis.

For methanol formation, the reaction pathway follows successive hydrogenation of formate to methoxy species adsorbed on the surface, followed by desorption of methoxy as methanol. Time-resolved evolution of adsorbed species was measured by in situ DRIFTS under CO_2_ hydrogenation condition (Figure 5a,b). The complete DRIFTS spectra for all catalysts and detailed peak assignment are shown in Figures S12–S14 and Table S3. To check the possibility of methanol formation via CO hydrogenation, we performed in situ DRIFTS analysis under a CO + H_2_ environment (Figure S15), which resulted in no methoxy formation and confirmed the formate hydrogenation pathway for methanol formation. Formate species appeared rapidly at the start of the reaction along with carbonate and bicarbonate over all doped catalysts. Methoxy species appeared with a delay of about 10 min over Co–In–ZrO_2_ and In–ZrO_2_. Methoxy formation over Co–In–ZrO_2_ was faster in comparison to In–ZrO_2_. Methoxy species were not detected over Co–ZrO_2_. After 100 min, the flow of CO_2_ was stopped, and formate species reduced rapidly over Co–In–ZrO_2_ and Co–ZrO_2_. The rate of the disappearance of formate over In-ZrO_2_ was slower. Simultaneously, the abundance of methoxy increased marginally, as more sites for adsorption were available after stopping CO_2_. The rate of disappearance of methoxy over Co–In–ZrO_2_ was comparatively faster than that over In–ZrO_2_. These results indicate that formate adsorbed over the Co–Zr interface was more reactive, either to form methoxy or decompose to CO. In addition, the desorption of methoxy species adsorbed over the Co–In–ZrO_2_ surface was also facile.

Because methoxy species were not observed over the Co–ZrO_2_ catalyst, it is evident that hydrogen dissociation over In atoms promotes the hydrogenation of formate species adsorbed on Co–In–ZrO_2_. This cooperative effect was further confirmed by the temperature-dependent in situ DRIFTS study. Formate species appeared over the Co–In–ZrO_2_ catalyst at a temperature as low as 100 °C, followed by Co–ZrO_2_ and In–ZrO_2_ (Figure 5c). This result shows that In atoms promote the hydrogenation of CO_2_ adsorbed over Co–Zr sites to formate. The peaks for the adsorbed methoxy species also appeared at a lower temperature over Co–In–ZrO_2_ than over In–ZrO_2_ (Figure 5d). Therefore, the separation of sites for CO_2_ adsorption and H_2_ dissociation over Co–In–ZrO_2_ promotes hydrogenation of adsorbed species to yield methanol.

### Theoretical Confirmation of the Cooperative Effect

**Figure 6:**
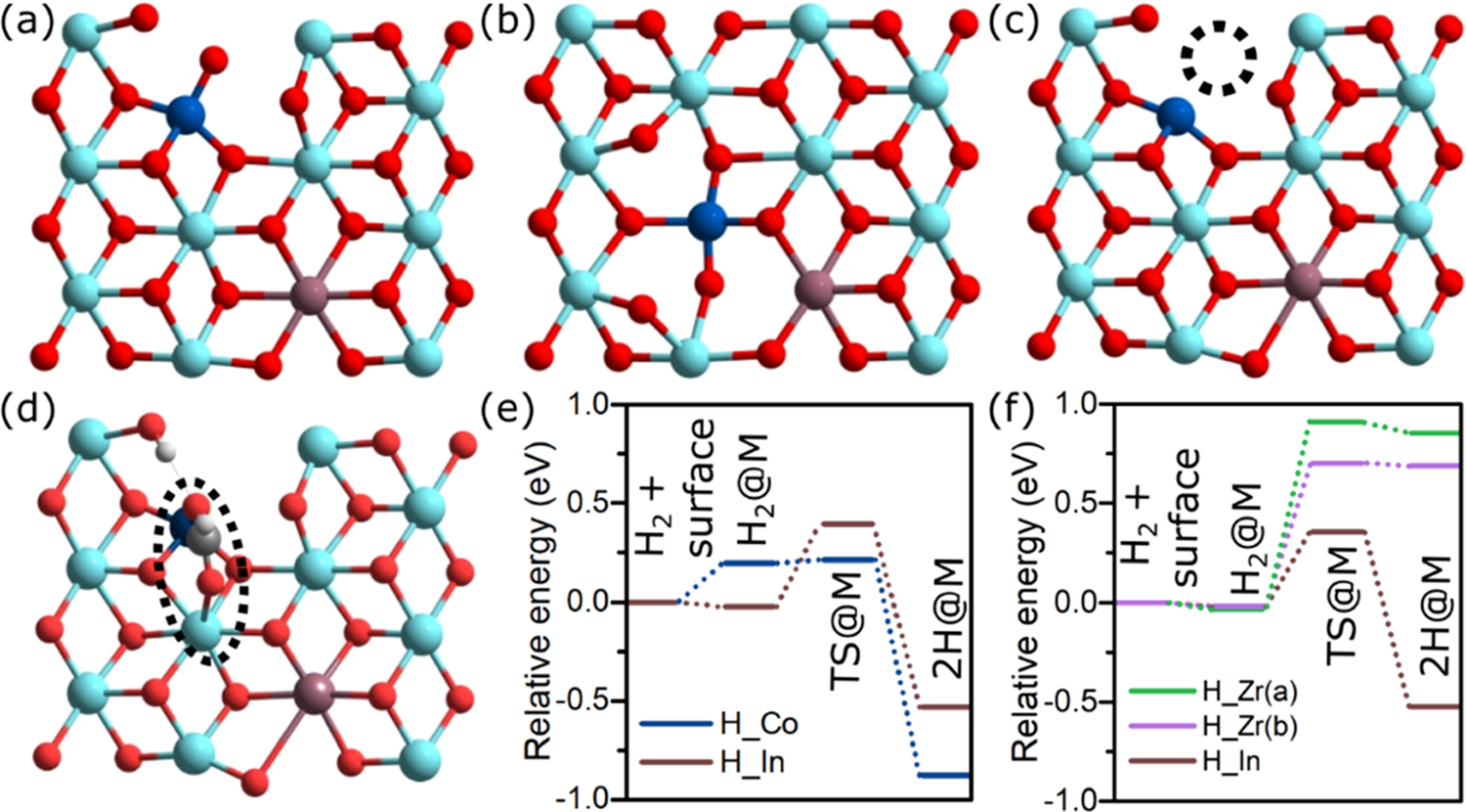
Optimized geometries of Co- and In-doped ZrO_2_ (blue, cyan, and brown correspond to Co, Zr, and In, respectively.) with (a) Co and In atoms far apart and (b) adjacent to each other. (c) Optimized geometry of Co–In–ZrO_2_ having oxygen vacancy near the Co atom (the dotted circle represents the original position of removed oxygen). (d) Geometry of the catalyst with formate stabilized at the Co–Zr interface, used to calculate H_2_ dissociation energy. (e) H_2_ dissociation over Co and In atoms over the surface of Co- and In-doped ZrO_2_ having oxygen vacancy shown in (c). (f) H_2_ dissociation over the In atom and two Zr atoms over the surface of Co- and In-doped ZrO_2_ having formate species stabilized at the Co–Zr interface shown in Figure 6d. The detailed structures for H_2_ dissociation steps including the transition state are shown in the Supporting Information (Figures S18–S19).

Density functional theory (DFT) calculation was used to investigate the impact of dual-atom (In and Co) doping on the geometry of the Co–In–ZrO_2_ surface and the feasibility of H_2_ dissociation over Co and In atoms. The (101) facet of *t-*ZrO_2_ was selected as the computational model, because it was the only phase observed in XRD analysis. The ratio of Co/In/Zr in the model was 1:1:10, which is similar to the experimentally observed value of 1:1:7 at the surface. To obtain the geometry of Co–In–ZrO_2_, we utilized the stable geometry of *t*-ZrO_2_ as the initial structure and screened possible sites for In and Co atoms on the first and second layers. Figure S16 shows the optimized geometry for different positions of In and Co atoms in the Co–In-doped ZrO_2_(101) surface. Most stable structures were obtained when both In and Co atoms were on the first layer (Figure 6a,b). The energy difference between the structure with In and Co atoms adjacent to each other (Figure 6b) and with In and Co separated from each other (Figure 6a) was only 0.07 eV. This corroborates the experimental observation of atomic dispersion of Co and In atoms in the ZrO_2_ matrix leading to a random arrangement of Co and In atoms in proximity. The surface distortion of ZrO_2_ with doped In was smaller than the distortion with the doping of Co. Co atoms were coordinated with 5 oxygen atoms, resulting in an imbalance in coordination number between cations.

Using the most stable structure shown in Figure 6a, oxygen vacancy formation on the surface of Co–In–ZrO_2_ was investigated. Seven possible sites near the doped Co and In atoms were considered (Figure S17). Oxygen vacancies adjacent to Co atoms were comparatively easier to form because of the difference in the coordination between Co and Zr atoms. The most stable structure was with oxygen vacancy adjacent to the Co atom, as depicted in Figure 6c. A cavity was generated near the Co atom, which could serve as an active site for the CO_2_ and H_2_ activation.

Next, we considered the dissociation of H_2_ over the In and Co sites of Co–In–ZrO_2_ in the absence and presence of adsorbed formate species at the Co–Zr interface as shown in Figure 6d. On a clean surface, H_2_ dissociation was exothermic over both Co and In sites (Figure 6e). Therefore, the first H_2_ dissociation, responsible for the formation of formate species, was possible over both Co and In sites, with a barrier of 0.22 and 0.42 eV, respectively. H_2_ dissociation over Co was more exothermic with a lower activation energy likely due to the presence of undercoordinated O atoms nearby Co that would accept H^+^ during the heterolytic dissociation of H_2_.^61^ This is in accordance with the experimental results because formate easily forms over the surface of single-atom Co–ZrO_2_ catalysts. Next, we investigated the influence of adsorbed formate species on the Co site. The geometry of formate-adsorbed Co–In–ZrO_2_ was based on experimental results having the formate species coordinated with Co and Zr atoms (Figure 6d). Using this structure, H_2_ dissociation was not possible over the Co site with the adsorbed formate species. H_2_ dissociation over the In site was favorable with a barrier of 0.38 eV (Figure 6f). H_2_ dissociation over Zr atoms was endothermic, as expected. The proximity of In to the adsorbed formate species would facilitate transfer of hydrogen dissociated over In atoms to formate species, resulting in further hydrogenation of formate to methanol.

### Mechanism

**Figure 7:**
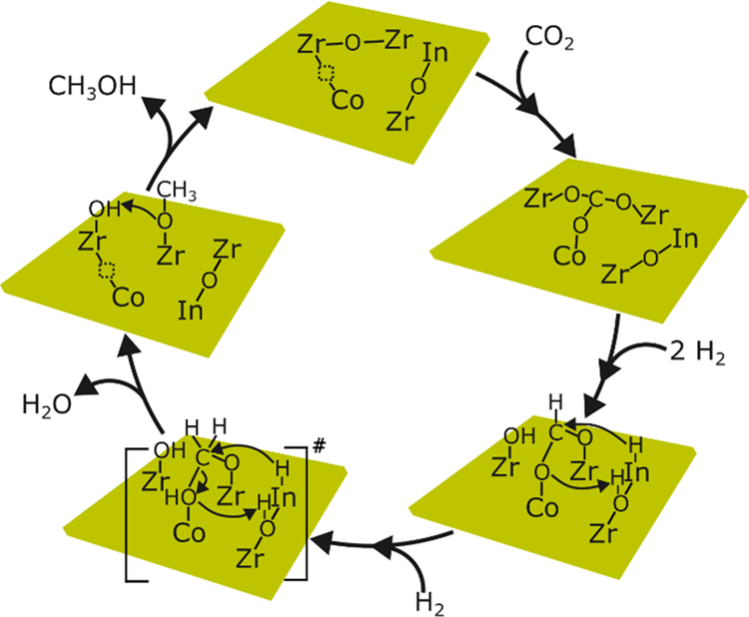
Schematic representation of the proposed mechanism for CO_2_ hydrogenation to methanol over the ternary Co–In–ZrO_2_ showing the site separation of CO_2_ adsorption and H_2_ dissociation over Co and In sites, respectively.

Based on the above results, we propose the following mechanism for CO_2_ hydrogenation to methanol over the Co–In–ZrO_2_ catalyst through the cooperative effect of Co and In (Figure 7). Doping of Co^2+^ creates an oxygen-vacant site for the adsorption of CO_2_. First, H_2_ dissociation might happen on either Co or In atoms for hydrogenation of adsorbed CO_2_, although the presence of In promotes hydrogenation of adsorbed CO_2_ to formate. Following formate formation, the ability of Co to dissociate H_2_ and hydrogenate formate is hindered. Instead, H_2_ dissociated over adjacent In atoms facilitates the hydrogenation of formate to methoxy species and promotes the desorption of methoxy species as methanol. Over the dual-atom catalyst, because formate gets selectively hydrogenated to methoxy by the cooperation of Co and In atoms, both the selectivity and productivity of methanol increase. Thus, the presence of a Co single atom reduces catalyst poisoning by strong adsorption of CO_2_ and formate. In atoms assisted in H_2_ dissociation and hydrogenation of adsorbed intermediates. Consequently, a higher methanol selectivity can be obtained even at a low H_2_ partial pressure.

### Generalizing Dual-Atom Co-Containing Catalysts

**Figure 8:**
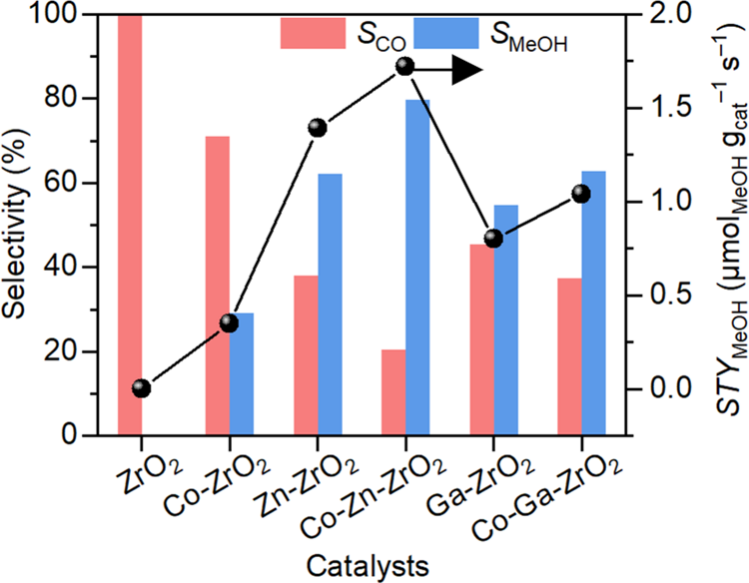
Comparison of the catalytic performance of Co–Zn–ZrO_2_ and Co–Ga–ZrO_2_ with their single-atom-doped oxide counterparts. Reaction conditions: 300 °C, 3 MPa, 30,000 mL h^–1^ g_cat_^–1^, H_2_/CO_2_ = 4:1. The line for STY_MeOH_ acts as visual guidance.

To evaluate the generality of the idea of mitigating the poisonous effect by introducing a Co single atom, we prepared Co–Zn–ZrO_2_ and Co–Ga–ZrO_2_ dual-atom oxides for CO_2_ hydrogenation to methanol. In both cases, the methanol selectivity and methanol STY increased compared to the single-atom doped oxides (Figure 8). Characterization of the catalysts and investigation of reaction mechanism for Co–Zn–ZrO_2_ and Co–Ga–ZrO_2_ revealed a similar mode of action for increased methanol formation by separation of sites for CO_2_ adsorption and H_2_ dissociation (see Supporting Information Sections 3 and 4). The above results establish that this strategy can be effectively used to enhance the methanol selectivity and yield using Co single atoms to create an active site for the adsorption of CO_2_ and stabilization of intermediates.

## Conclusions

During the hydrogenation of CO_2_ to methanol, it is essential to maintain high selectivity while increasing the methanol yield. Improving the reactivity of the mixed-oxide catalyst requires countering the poisoning effect of the strongly adsorbed CO_2_ and formate species, which reduces the H_2_ dissociation ability. Counter to the strategy reported so far of introducing metal nanoparticles to dissociate H_2_, we demonstrate that the separation of the adsorption sites for CO_2_ and H_2_ over a dual-atom mixed-oxide catalyst having a Co single atom is an effective way to promote both the selectivity and productivity of methanol by mitigating the poisonous effect. We prepared a Co- and In-doped ZrO_2_ (Co–In–ZrO_2_) catalyst where Co and In atoms were atomically dispersed. Structural characterization indicated the atomic distribution of Co and In atoms. Co–In–ZrO_2_ showed better methanol selectivity and productivity than the corresponding single-atom-doped oxides. Kinetic analysis showed that Co–In–ZrO_2_ had the lowest apparent activation energy for methanol formation, with a concomitant highest apparent activation energy for CO formation. It was found that Co–In–ZrO_2_ was less dependent on the H_2_ and CO_2_ partial pressure than were binary oxides during methanol formation. Co sites were responsible for the CO_2_ adsorption and formate formation. In sites were responsible for H_2_ dissociation and promoted formate hydrogenation to methoxy and methoxy desorption as methanol selectively. This cooperative effect was not limited to the Co–In system and was also observed in Co–Zn and Co–Ga systems. This study shows a different approach to selective catalyst design based on the preferential adsorption of substrates and intermediates. We believe that this strategy will help in the design of selective catalysts for complicated reactions involving multiple substrates and intermediates.

## Supporting Information Available

Methods, additional data for the characterization of the catalysts, the elucidation of reaction mechanism, and the theoretical calculation, as well as an additional discussion of characterization and catalytic activity of Co–Zn–ZrO_2_ and Co–Ga–ZrO_2_ catalysts (PDF)

## References

[ref1] Jiang X.; Nie X.; Guo X.; Song C.; Chen J. G. Recent Advances in Carbon Dioxide Hydrogenation to Methanol via Heterogeneous Catalysis. Chem. Rev. 2020, 120 (15), 7984–8034. 10.1021/acs.chemrev.9b00723. 32049507

[ref2] Zhong J.; Yang X.; Wu Z.; Liang B.; Huang Y.; Zhang T. State of the Art and Perspectives in Heterogeneous Catalysis of CO_2_ Hydrogenation to Methanol. Chem. Soc. Rev. 2020, 49, 1385–1413. 10.1039/C9CS00614A. 32067007

[ref3] Ra E. C.; Kim K. Y.; Kim E. H.; Lee H.; An K.; Lee J. S. Recycling Carbon Dioxide through Catalytic Hydrogenation: Recent Key Developments and Perspectives. ACS Catal. 2020, 10 (19), 11318–11345. 10.1021/acscatal.0c02930.

[ref4] Bao J.; Yang G.; Yoneyama Y.; Tsubaki N. Significant Advances in C1 Catalysis: Highly Efficient Catalysts and Catalytic Reactions. ACS Catal. 2019, 9 (4), 3026–3053. 10.1021/acscatal.8b03924.

[ref5] Olah G. A.; Goeppert A.; Prakash G. K. S. Beyond Oil and Gas: The Methanol Economy; Wiley, 2009.

[ref6] Roy S.; Cherevotan A.; Peter S. C. Thermochemical CO_2_ Hydrogenation to Single Carbon Products: Scientific and Technological Challenges. ACS Energy Lett. 2018, 3 (8), 1938–1966. 10.1021/acsenergylett.8b00740.

[ref7] Porosoff M. D.; Yan B.; Chen J. G. Catalytic Reduction of CO_2_ by H_2_ for Synthesis of CO, Methanol and Hydrocarbons: Challenges and Opportunities. Energy Environ. Sci. 2016, 9, 62–73. 10.1039/C5EE02657A.

[ref8] De S.; Dokania A.; Ramirez A.; Gascon J. Advances in the Design of Heterogeneous Catalysts and Thermocatalytic Processes for CO_2_ Utilization. ACS Catal. 2020, 10 (23), 14147–14185. 10.1021/acscatal.0c04273.

[ref9] Jangam A.; Das S.; Dewangan N.; Hongmanorom P.; Hui W. M.; Kawi S. Conversion of CO_2_ to C1 Chemicals: Catalyst Design, Kinetics and Mechanism Aspects of the Reactions. Catal. Today 2020, 358, 3–29. 10.1016/j.cattod.2019.08.049.

[ref10] Martin O.; Martín A. J.; Mondelli C.; Mitchell S.; Segawa T. F.; Hauert R.; Drouilly C.; Curulla-Ferré D.; Pérez-Ramírez J. Indium Oxide as a Superior Catalyst for Methanol Synthesis by CO_2_ Hydrogenation. Angew. Chem., Int. Ed. 2016, 55 (21), 6261–6265. 10.1002/anie.201600943. 26991730

[ref11] Wang J.; Zhang G.; Zhu J.; Zhang X.; Ding F.; Zhang A.; Guo X.; Song C. CO_2_ Hydrogenation to Methanol over In_2_O_3_-Based Catalysts: From Mechanism to Catalyst Development. ACS Catal. 2021, 11 (3), 1406–1423. 10.1021/acscatal.0c03665.

[ref12] Feng W. H.; Yu M. M.; Wang L. J.; Miao Y. T.; Shakouri M.; Ran J.; Hu Y.; Li Z.; Huang R.; Lu Y. L.; Gao D.; Wu J. F. Insights into Bimetallic Oxide Synergy during Carbon Dioxide Hydrogenation to Methanol and Dimethyl Ether over GaZrO_x_ Oxide Catalysts. ACS Catal. 2021, 11 (8), 4704–4711. 10.1021/acscatal.0c05410.

[ref13] Tada S.; Ochiai N.; Kinoshita H.; Yoshida M.; Shimada N.; Joutsuka T.; Nishijima M.; Honma T.; Yamauchi N.; Kobayashi Y.; Iyoki K. Active Sites on Zn_x_Zr_1–x_O_2–x_ Solid Solution Catalysts for CO_2_-to-Methanol Hydrogenation. ACS Catal. 2022, 12, 7748–7759. 10.1021/acscatal.2c01996.

[ref14] Wang J.; Li G.; Li Z.; Tang C.; Feng Z.; An H.; Liu H.; Liu T.; Li C. A Highly Selective and Stable ZnO-ZrO_2_ Solid Solution Catalyst for CO_2_ Hydrogenation to Methanol. Sci. Adv. 2017, 3, e1701290 10.1126/sciadv.1701290. 28989964 PMC5630239

[ref15] Wang J.; Tang C.; Li G.; Han Z.; Li Z.; Liu H.; Cheng F.; Li C. High-Performance MaZrO_x_ (Ma = Cd, Ga) Solid-Solution Catalysts for CO_2_ Hydrogenation to Methanol. ACS Catal. 2019, 9 (11), 10253–10259. 10.1021/acscatal.9b03449.

[ref16] Sha F.; Tang C.; Tang S.; Wang Q.; Han Z.; Wang J.; Li C. The Promoting Role of Ga in ZnZrO_x_ Solid Solution Catalyst for CO_2_ Hydrogenation to Methanol. J. Catal. 2021, 404, 383–392. 10.1016/j.jcat.2021.09.030.

[ref17] Ye J.; Liu C.; Mei D.; Ge Q. Active Oxygen Vacancy Site for Methanol Synthesis from CO_2_ Hydrogenation on In_2_O_3_(110): A DFT Study. ACS Catal. 2013, 3 (6), 1296–1306. 10.1021/cs400132a.

[ref18] Frei M. S.; Mondelli C.; Cesarini A.; Krumeich F.; Hauert R.; Stewart J. A.; Curulla Ferré D.; Pérez-Ramírez J. Role of Zirconia in Indium Oxide-Catalyzed CO_2_ Hydrogenation to Methanol. ACS Catal. 2020, 10 (2), 1133–1145. 10.1021/acscatal.9b03305.

[ref19] Tsoukalou A.; Abdala P. M.; Armutlulu A.; Willinger E.; Fedorov A.; Müller C. R. Operando X-Ray Absorption Spectroscopy Identifies a Monoclinic ZrO_2_:In Solid Solution as the Active Phase for the Hydrogenation of CO_2_ to Methanol. ACS Catal. 2020, 10 (17), 10060–10067. 10.1021/acscatal.0c01968.

[ref20] Tsoukalou A.; Serykh A. I.; Willinger E.; Kierzkowska A.; Abdala P. M.; Fedorov A.; Müller C. R. Hydrogen Dissociation Sites on Indium-Based ZrO_2_-Supported Catalysts for Hydrogenation of CO_2_ to Methanol. Catal. Today 2022, 387, 38–46. 10.1016/j.cattod.2021.04.010.

[ref21] Kattel S.; Yan B.; Yang Y.; Chen J. G.; Liu P. Optimizing Binding Energies of Key Intermediates for CO_2_ Hydrogenation to Methanol over Oxide-Supported Copper. J. Am. Chem. Soc. 2016, 138 (38), 12440–12450. 10.1021/jacs.6b05791. 27571313

[ref22] Jung K. D.; Bell A. T. Role of Hydrogen Spillover in Methanol Synthesis over Cu/ZrO_2_. J. Catal. 2000, 193 (2), 207–223. 10.1006/jcat.2000.2881.

[ref23] Tang C.; Tang S.; Sha F.; Han Z.; Feng Z.; Wang J.; Li C. Insights into the Selectivity Determinant and Rate-Determining Step of CO_2_ Hydrogenation to Methanol. J. Phys. Chem. C 2022, 126 (25), 10399–10407. 10.1021/acs.jpcc.2c02995.

[ref24] Polierer S.; Jelic J.; Pitter S.; Studt F. On the Reactivity of the Cu/ZrO_2_ System for the Hydrogenation of CO_2_ to Methanol: A Density Functional Theory Study. J. Phys. Chem. C 2019, 123 (44), 26904–26911. 10.1021/acs.jpcc.9b06500.

[ref25] Chou C. Y.; Lobo R. F. Direct Conversion of CO_2_ into Methanol over Promoted Indium Oxide-Based Catalysts. Appl. Catal., A 2019, 583, 117144 10.1016/j.apcata.2019.117144.

[ref26] Sha F.; Tang S.; Tang C.; Feng Z.; Wang J.; Li C. The Role of Surface Hydroxyls on ZnZrO_x_ Solid Solution Catalyst in CO_2_ Hydrogenation to Methanol. Chin. J. Catal. 2023, 45, 162–173. 10.1016/S1872-2067(22)64176-7.

[ref27] Šot P.; Noh G.; Weber I. C.; Pratsinis S. E.; Copéret C. The Influence of ZnO–ZrO_2_ Interface in Hydrogenation of CO_2_ to CH_3_OH. Helv. Chim. Acta 2022, 105 (3), e202200007 10.1002/hlca.202200007.

[ref28] Chen T. Y.; Cao C.; Chen T. B.; Ding X.; Huang H.; Shen L.; Cao X.; Zhu M.; Xu J.; Gao J.; Han Y. F. Unraveling Highly Tunable Selectivity in CO_2_ Hydrogenation over Bimetallic In-Zr Oxide Catalysts. ACS Catal. 2019, 9 (9), 8785–8797. 10.1021/acscatal.9b01869.

[ref29] Fang H.; Zhao G.; Cheng D.; Liu J.; Lan D.; Jiang Q.; Liu X.; Ge J.; Xu Z.; Xu H. MOF-Derived Bimetallic Core–Shell Catalyst HZSM-5@ZrO_2_–In_2_O_3_: High CO_2_ Conversion in Reverse Water Gas Shift Reaction. Mater. Chem. Front. 2022, 6, 2826–2834. 10.1039/D2QM00307D.

[ref30] Frei M. S.; Mondelli C.; García-Muelas R.; Kley K. S.; Puértolas B.; López N.; Safonova O. V.; Stewart J. A.; Curulla Ferré D.; Pérez-Ramírez J. Atomic-Scale Engineering of Indium Oxide Promotion by Palladium for Methanol Production via CO_2_ Hydrogenation. Nat. Commun. 2019, 10 (1), 3377 10.1038/s41467-019-11349-9. 31358766 PMC6662860

[ref31] Araújo T. P.; Mondelli C.; Agrachev M.; Zou T.; Willi P. O.; Engel K. M.; Grass R. N.; Stark W. J.; Safonova O. V.; Jeschke G.; Mitchell S.; Pérez-Ramírez J. Flame-Made Ternary Pd-In_2_O_3_-ZrO_2_ Catalyst with Enhanced Oxygen Vacancy Generation for CO_2_ Hydrogenation to Methanol. Nat. Commun. 2022, 13 (1), 5610 10.1038/s41467-022-33391-w. 36153333 PMC9509363

[ref32] Frei M. S.; Mondelli C.; García-Muelas R.; Morales-Vidal J.; Philipp M.; Safonova O. V.; López N.; Stewart J. A.; Ferré D. C.; Pérez-Ramírez J. Nanostructure of Nickel-Promoted Indium Oxide Catalysts Drives Selectivity in CO_2_ Hydrogenation. Nat. Commun. 2021, 12 (1), 1960 10.1038/s41467-021-22224-x. 33785755 PMC8010022

[ref33] Song L.; Wang H.; Wang S.; Qu Z. Dual-Site Activation of H_2_ over Cu/ZnAl_2_O_4_ Boosting CO_2_ Hydrogenation to Methanol. Appl. Catal., B 2023, 322, 122137 10.1016/j.apcatb.2022.122137.

[ref34] Bavykina A.; Yarulina I.; Al Abdulghani A. J.; Gevers L.; Hedhili M. N.; Miao X.; Galilea A. R.; Pustovarenko A.; Dikhtiarenko A.; Cadiau A.; Aguilar-Tapia A.; Hazemann J.-L.; Kozlov S. M.; Oud-Chikh S.; Cavallo L.; Gascon J. Turning a Methanation Co Catalyst into an In–Co Methanol Producer. ACS Catal. 2019, 9 (8), 6910–6918. 10.1021/acscatal.9b01638.

[ref35] Li L.; Yang B.; Gao B.; Wang Y.; Zhang L.; Ishihara T.; Qi W.; Guo L. CO_2_ Hydrogenation Selectivity Shift over In-Co Binary Oxides Catalysts: Catalytic Mechanism and Structure-Property Relationship. Chin. J. Catal. 2022, 43 (3), 862–876. 10.1016/S1872-2067(21)63870-6.

[ref36] Zhu J.; Cannizzaro F.; Liu L.; Zhang H.; Kosinov N.; Filot I. A. W.; Rabeah J.; Brückner A.; Hensen E. J. M. Ni-In Synergy in CO_2_ Hydrogenation to Methanol. ACS Catal. 2021, 11 (18), 11371–11384. 10.1021/acscatal.1c03170. 34557327 PMC8453486

[ref37] Han X.; Xiao T.; Li M.; Hao Z.; Chen J.; Pan Y.; Zi X.; Zhang H.; Xi S.; Wai H. M.; Kawi S.; Ma X. Synergetic Interaction between Single-Atom Cu and Ga_2_O_3_ Enhances CO_2_ Hydrogenation to Methanol over CuGaZrO_x_. ACS Catal. 2023, 13 (20), 13679–13690. 10.1021/acscatal.3c03431.

[ref38] Lee K.; Anjum U.; Araújo T. P.; Mondelli C.; He Q.; Furukawa S.; Pérez-Ramírez J.; Kozlov S. M.; Yan N. Atomic Pd-Promoted ZnZrO_x_ Solid Solution Catalyst for CO_2_ Hydrogenation to Methanol. Appl. Catal., B 2022, 304, 120994 10.1016/j.apcatb.2021.120994.

[ref39] Lee K.; Mendes P. C. D.; Jeon H.; Song Y.; Dickieson M. P.; Anjum U.; Chen L.; Yang T. C.; Yang C. M.; Choi M.; Kozlov S. M.; Yan N. Engineering Nanoscale H Supply Chain to Accelerate Methanol Synthesis on ZnZrO_x_. Nat. Commun. 2023, 14 (1), 819 10.1038/s41467-023-36407-1. 36781851 PMC9925737

[ref40] Sun K.; Rui N.; Zhang Z.; Sun Z.; Ge Q.; Liu C. J. A Highly Active Pt/In_2_O_3_ catalyst for CO_2_ Hydrogenation to Methanol with Enhanced Stability. Green Chem. 2020, 22 (15), 5059–5066. 10.1039/D0GC01597K.

[ref41] Xu D.; Hong X.; Liu G. Highly Dispersed Metal Doping to ZnZr Oxide Catalyst for CO_2_ Hydrogenation to Methanol: Insight into Hydrogen Spillover. J. Catal. 2021, 393, 207–214. 10.1016/j.jcat.2020.11.039.

[ref42] Dostagir N. H. M.; Thompson C.; Kobayashi H.; Karim A. M.; Fukuoka A.; Shrotri A. Rh Promoted In_2_O_3_ as a Highly Active Catalyst for CO_2_ Hydrogenation to Methanol. Catal. Sci. Technol. 2020, 10 (24), 8196–8202. 10.1039/D0CY01789B.

[ref43] Li M. M.; Zou H.; Zheng J.; Wu T.; Chan T.; Soo Y.; Wu X.; Gong X.; Chen T.; Roy K.; Held G.; Tsang S. C. E. Methanol Synthesis at a Wide Range of H_2_/CO_2_ Ratios over a Rh-In Bimetallic Catalyst. Angew. Chem. 2020, 132 (37), 16173–16180. 10.1002/ange.202000841. 32458500

[ref44] Rui N.; Wang X.; Deng K.; Moncada J.; Rosales R.; Zhang F.; Xu W.; Waluyo I.; Hunt A.; Stavitski E.; Senanayake S. D.; Liu P.; Rodriguez J. A. Atomic Structural Origin of the High Methanol Selectivity over In_2_O_3_-Metal Interfaces: Metal-Support Interactions and the Formation of a InO_x_ Overlayer in Ru/In_2_O_3_ Catalysts during CO_2_ Hydrogenation. ACS Catal. 2023, 13, 3187–3200. 10.1021/acscatal.2c06029.

[ref45] Shen C.; Sun K.; Zou R.; Wu Q.; Mei D.; Liu C. J. CO_2_ Hydrogenation to Methanol on Indium Oxide-Supported Rhenium Catalysts: The Effects of Size. ACS Catal. 2022, 12 (20), 12658–12669. 10.1021/acscatal.2c03709.

[ref46] Jia X.; Sun K.; Wang J.; Shen C.; Liu C. jun. Selective Hydrogenation of CO_2_ to Methanol over Ni/In_2_O_3_ Catalyst. J. Energy Chem. 2020, 50, 409–415. 10.1016/j.jechem.2020.03.083.

[ref47] Wang J.; Sun K.; Jia X.; Liu C. jun. CO_2_ Hydrogenation to Methanol over Rh/In_2_O_3_ Catalyst. Catal. Today 2021, 365, 341–347. 10.1016/j.cattod.2020.05.020.

[ref48] Rui N.; Wang Z.; Sun K.; Ye J.; Ge Q.; Liu C. jun. CO_2_ Hydrogenation to Methanol over Pd/In_2_O_3_: Effects of Pd and Oxygen Vacancy. Appl. Catal., B 2017, 218, 488–497. 10.1016/j.apcatb.2017.06.069.

[ref49] Lu R.; Zhang X.; Li S.-S.; Wang Y.; Song T.; Zhu T.; Zhang H.; Zheng R.; Zhang X. Pt-Promoted In_2_O_3_ Reduction and Reconstruction at the Pt/In_2_O_3_ Interface in CO_2_ Hydrogenation Atmosphere. ChemCatChem 2023, 15, e202201435 10.1002/cctc.202201435.

[ref50] Cannizzaro F.; Hensen E. J. M.; Filot I. A. W. The Promoting Role of Ni on In_2_O_3_ for CO_2_ Hydrogenation to Methanol. ACS Catal. 2023, 13, 1875–1892. 10.1021/acscatal.2c04872. 36776383 PMC9903295

[ref51] Li C. S.; Melaet G.; Ralston W. T.; An K.; Brooks C.; Ye Y.; Liu Y. S.; Zhu J.; Guo J.; Alayoglu S.; Somorjai G. A. High-Performance Hybrid Oxide Catalyst of Manganese and Cobalt for Low-Pressure Methanol Synthesis. Nat. Commun. 2015, 6 (1), 6538 10.1038/ncomms7538. 25754475

[ref52] Pustovarenko A.; Dikhtiarenko A.; Bavykina A.; Gevers L.; Ramírez A.; Russkikh A.; Telalovic S.; Aguilar A.; Hazemann J. L.; Ould-Chikh S.; Gascon J. Metal-Organic Framework-Derived Synthesis of Cobalt Indium Catalysts for the Hydrogenation of CO_2_ to Methanol. ACS Catal. 2020, 10 (9), 5064–5076. 10.1021/acscatal.0c00449.

[ref53] Shannon R. D. Revised Effective Ionic Radii and Systematic Studies of Interatomic Distances in Halides and Chalcogenides. Acta Crystallogr., Sect. A 1976, 32 (5), 751–767. 10.1107/S0567739476001551.

[ref54] Ye J.; Liu C.; Mei D.; Ge Q. Methanol Synthesis from CO_2_ Hydrogenation over a Pd_4_/In_2_O_3_ Model Catalyst: A Combined DFT and Kinetic Study. J. Catal. 2014, 317, 44–53. 10.1016/j.jcat.2014.06.002.

[ref55] Yang C.; Pei C.; Luo R.; Liu S.; Wang Y.; Wang Z.; Zhao Z. J.; Gong J. Strong Electronic Oxide-Support Interaction over In_2_O_3_/ZrO_2_ for Highly Selective CO_2_ Hydrogenation to Methanol. J. Am. Chem. Soc. 2020, 142 (46), 19523–19531. 10.1021/jacs.0c07195. 33156989

[ref56] Dostagir N. H. M.; Rattanawan R.; Gao M.; Ota J.; Hasegawa J. Y.; Asakura K.; Fukouka A.; Shrotri A. Co Single Atoms in ZrO_2_ with Inherent Oxygen Vacancies for Selective Hydrogenation of CO_2_ to CO. ACS Catal. 2021, 11 (15), 9450–9461. 10.1021/acscatal.1c02041.

[ref57] Feng Z.; Tang C.; Zhang P.; Li K.; Li G.; Wang J.; Feng Z.; Li C. Asymmetric Sites on the ZnZrO_x_ Catalyst for Promoting Formate Formation and Transformation in CO_2_ Hydrogenation. J. Am. Chem. Soc. 2023, 145 (23), 12663–12672. 10.1021/jacs.3c02248. 37261391

[ref58] Pokrovski K.; Jung K. T.; Bell A. T. Investigation of CO and CO_2_ Adsorption on Tetragonal and Monoclinic Zirconia. Langmuir 2001, 17 (14), 4297–4303. 10.1021/la001723z.

[ref59] Korhonen S. T.; Calatayud M.; Krause A. O. I. Structure and Stability of Formates and Carbonates on Monoclinic Zirconia: A Combined Study by Density Functional Theory and Infrared Spectroscopy. J. Phys. Chem. C 2008, 112 (41), 16096–16102. 10.1021/jp803353v.

[ref60] Tsoukalou A.; Bushkov N. S.; Docherty S. R.; Mance D.; Serykh A. I.; Abdala P. M.; Copéret C.; Fedorov A.; Müller C. R. Surface Intermediates in In-Based ZrO_2_-Supported Catalysts for Hydrogenation of CO_2_ to Methanol. J. Phys. Chem. C 2022, 126 (4), 1793–1799. 10.1021/acs.jpcc.1c08814.

[ref61] Aireddy D. R.; Ding K. Heterolytic Dissociation of H_2_ in Heterogeneous Catalysis. ACS Catal. 2022, 12 (8), 4707–4723. 10.1021/acscatal.2c00584.

