# Supplementary material for: Mitigating the Poisoning Effect of Formate during CO2 Hydrogenation to Methanol over Co-Containing Dual-Atom Oxide Catalysts
Source: JACS Au. 2024 Feb 2;4(3):1048–58. doi: 10.1021/jacsau.3c00789 (PMC10976564; doi:10.1021/jacsau.3c00789)
Supplement: Supplementary file 1 — au3c00789_si_001.pdf [file au3c00789_si_001.pdf]

# Supporting Information

# Mitigating the poisoning effect of formate during $CO_2$ hydrogenation to methanol over Co containing dual-atom oxide catalysts

*Nazmul Hasan MD Dostagir,[a] Carlo Robert Tomuschat,[a,b] Kai Oshiro,[c] Min Gao,[d] Jun-ya Hasegawa,[a,e] Atsushi Fukuoka,[a]* and Abhijit Shrotri[a]**

[a] Institute for Catalysis, Hokkaido University, Kita 21 Nishi 10, Kita-ku, Sapporo, Hokkaido 001-0021, Japan

[b] Department of Chemistry, TUM School of Natural Sciences, Technical University of Munich, Lichtenbergstraße 4, 85748 Garching, Germany

[c] Graduate School of Chemical Sciences and Engineering, Hokkaido University, Kita 13 Nishi 8, Kita-ku, Hokkaido, 060-8628, Japan

[d] Institute for Chemical Reaction Design and Discovery, Hokkaido University, Kita 21 Nishi 10, Kita-ku, Sapporo, Hokkaido 001-0021, Japan

[e] Interdisciplinary Research Center for Catalytic Chemistry, National Institute of Advanced Industrial Science and Technology, Central 5, 1-1-1 Higashi, Tsukuba, Ibaraki 305-8565, Japan

* Email: ashrotri@cat.hokudai.ac.jp, fukuoka@cat.hokudai.ac.jp

# Table of Contents

**1. Methods** .......... **3**
**2. Additional Data for Co-In-$ZrO_2$ System** .......... **8**
2.1. Structural Characterization .......... 8
2.2. Catalytic Performance .......... 10
2.3. In situ DRIFTS .......... 14
**3. Supporting Discussion of Co-Zn-$ZrO_2$ and Co-Ga-$ZrO_2$ Systems** .......... **21**
3.1. Structural Characterization .......... 21
3.2. Catalytic Performance .......... 22
3.3. Investigation of Reaction Mechanism .......... 22
**4. Supporting Tables and Figures for Co-Zn-$ZrO_2$ and Co-Ga-$ZrO_2$ Systems** .......... **24**
4.1. Structural Characterization .......... 24
4.2. Catalytic Performance .......... 27
4.3. Investigation of Reaction Mechanism .......... 28
**5. Supplementary References** .......... **33**

# 1. Methods

**X-ray diffraction.** X-ray diffraction (XRD) patterns were measured with a Rigaku Ultima IV (Rigaku Corporation) diffractometer in Bragg-Brentano geometry using CuKα X-ray radiation ($\lambda$ = 1.54 Å) operating at 40 kV and 20 mA with a D-tex Ultra (Rigaku Corporation) silicon strip detector. The X-ray diffraction patterns were collected at ambient temperature and pressure.

**$N_2$ adsorption-desorption experiments.** $N_2$ adsorption-desorption isotherms were measured at −196 °C using a Belsorp Mini II analyzer (BEL Japan Inc.). Prior to the adsorption, all samples were degassed under vacuum at 120 °C for 2 h in a BELPREP VAC II (Microtrac Retsch GmbH). The surface area was calculated using the BET theory in the relative pressure range of 0.05 to 0.35 of the $N_2$ adsorption isotherm.[1]

**X-ray photoelectron spectroscopy.** X-ray photoelectron spectroscopy (XPS) was performed with a JEOL JPS-9010MC instrument (JEOL Ltd.). Charge correction was done by adjusting the adventitious carbon peak to 284.6 eV. Atomic surface concentrations were quantified based on the XPS peak areas after Shirley background subtraction.

**Scanning transmission electron microscopy.** STEM images were obtained on a JEOL JEM-ARM200F atomic resolution electron microscope (JEOL Ltd.) at an acceleration voltage of 200 kV equipped with EDS detector EX-24221M1G5T. Titan3 G2 60-300 operating at 300 kV was used for double aberration-corrected HAADF STEM analysis. The digital micrograph software (GATAN) was used for image and spectrum acquisition and processing.

**Hydrogen temperature-programmed reduction.** Temperature programmed reduction of catalysts was carried out in presence of an $H_2$/Ar gas mixture (5 Vol.-% $H_2$) in a BELCAT II catalyst analyzer (BEL Japan Inc.) equipped with a high resolution 4-element thermal conductivity detector. Prior to the measurement, catalysts were pretreated at 200 °C for 1 hour under Ar flow. Measurements were done at a total flow rate of 50 mL min$^{-1}$ with a ramp rate of 10 °C min$^{-1}$.

**$CO_2$ Temperature-programmed desorption.** $CO_2$ TPD experiments were performed on a BELCAT II catalyst analyzer (BEL Japan Inc.) equipped with a high resolution 4-element thermal conductivity detector. Prior to the measurement, the samples were pretreated under Ar at 340 °C for 30 min followed by $CO_2$ adsorption at room temperature for 30 min. After purging with He for 30 min. TPD was performed with a ramp rate of 10 °C min$^{-1}$.

**Temperature-programmed formic acid decomposition.** Temperature programmed formic acid decomposition studies were conducted on a BELCAT II catalyst analyzer (BEL Japan Inc.) connected to a BELMASS online gas analyzer (BEL Japan Inc.) based on a systemized quadrupole mass spectrometer. For the sample preparation, the catalyst powder (100 mg) was suspended in *n*-hexane and 50 μL of formic acid were added. The solvent was removed, and the wet powder was washed with *n*-hexane. The sample was dried at 100 °C for one hour and transferred into the sample tube. The tube was heated to 700 °C with a heating rate of 10 °C min$^{-1}$ in a stream of He at a flow rate of 30 mL min$^{-1}$.

**Diffuse reflectance infrared Fourier transformation spectroscopy.** DRIFTS experiments were performed using a Perkin Elmer Spectrum 100 FTIR spectrometer (PerkinElmer, Inc.)

equipped with a MCT detector cooled with liquid $N_2$ (Figure S13). The powder catalyst was placed in an alumina sample holder and put into the measurement cell equipped with a heating unit and $CaF_2$ window. The catalyst was first pretreated under He at 300 °C for 30 min. A background spectrum was recorded under He atmosphere before introducing reactant gases. Final IR spectra were obtained by averaging eight scans (spectral resolution 4 cm$^{-1}$) and subtracting the background spectrum of the catalyst under He atmosphere.

**Determination of catalytic performance metrics.** $CO_2$ conversion, selectivity of CO and MeOH, and space-time yields (*STY*s) were calculated using the following equations:

$CO_2$ conversion ($X_{CO_2}$) in percent:

$$X_{CO_2} = \left( \frac{n_{CO(out)} + n_{MeOH(out)} + n_{CH_4(out)}}{n_{CO_2(out)} + n_{CO(out)} + n_{MeOH(out)} + n_{CH_4(out)}} \right) \times 100 \quad (2)$$

Selectivity of CO ($S_{CO}$), MeOH ($S_{MeOH}$) and $CH_4$ ($S_{CH_4}$) in percent:

$$S_{CO} = \left( \frac{n_{CO(out)}}{n_{CO(out)} + n_{MeOH(out)} + n_{CH_4(out)}} \right) \times 100 \quad (3)$$

$$S_{MeOH} = \left( \frac{n_{MeOH(out)}}{n_{CO(out)} + n_{MeOH(out)} + n_{CH_4(out)}} \right) \times 100 \quad (4)$$

$$S_{CH_4} = \left( \frac{n_{CH_4(out)}}{n_{CO(out)} + n_{MeOH(out)} + n_{CH_4(out)}} \right) \times 100 \quad (5)$$

Space time yield of product ($STY_{Product}$):

$$STY_{CO} = \left(\frac{GHSV \times [CO_2] \times X_{CO_2} \times S_{CO}}{22.4 \times 3.6}\right) \quad (6)$$

$$STY_{MeOH} = \left(\frac{GHSV \times [CO_2] \times X_{CO_2} \times S_{MeOH}}{22.4 \times 3.6}\right) \quad (7)$$

$n_{CO_2(out)}$, $n_{CO(out)}$, $n_{MeOH(out)}$, $n_{CH_4(out)}$ are the amount of substance of $CO_2$, CO, MeOH and $CH_4$ in the outlet stream calculated from GC analysis. *GHSV* is the gas-hourly space velocity in mL $h^{-1}$ $g_{cat}^{-1}$, [$CO_2$] is the concentration of $CO_2$ present in the feed gas mixture in Vol.-%. *STY* is reported in $\mu mol_{Product}$ $g_{cat}^{-1}$ $s^{-1}$.

**Density Functional Theory Calculation.**

Density functional theory (DFT) calculations were performed using the Vienna ab initio simulation package (VASP)[2] and the Generalized Gradient Approximation (GGA)[3] with the Perdew-Burke-Ernzerhof (PBE) functional. To account for the effect of electron correlation, the DFT + U approach was employed with a value of U = 7.0 eV. The lattice parameters of tetragonal $ZrO_2$ were calculated to be a = b = 3.66 Å and c = 5.19 Å, which agreed well with experimental results (a = b = 3.64 Å and c = 5.27 Å).[4] The $ZrO_2$(101) surface was modeled using a (2 × 3 × 2) element unit cell with a vacuum spacing of 16 Å to separate the slabs. All the calculated surface included 3 layers with the bottom layer fixed to represent the bulk state and the other layers fully relaxed. Converged results for total energies and forces were obtained using an energy cut-off of 400 eV and Gamma k-point grid. The threshold for self-consistent field (SCF) convergence was set to $10^{-6}$ eV, and all calculations were spin-polarized. The

transition state were done by inhouse program using optimizer implemented in GRRM17[5,6] and the electronic energy and gradient calculated by VASP.

Calculations at DFT-D3 level and DFT level were compared for $H_2$ dissociation process on formate adsorbed structure (Figure 6d and 6f in main text). DFT-D3 and DFT gave very similar results (energy of DFT level is shown in parentheses below). The barriers for $H_2$ dissociation ($E_{TS}$-$E_{H2ads}$, $E_{TS}$ is the electronic energy of transition state, $E_{H2ads}$ is the electronic energy of adsorbed $H_2$ molecule) were 0.35 eV (0.36 eV) on In atom, 0.87 eV (0.94 eV) on Zr(a), 0.65 eV (0.72 eV) on Zr(b) site@ formate stabilized at the Co-Zr interface. Therefore, results of DFT level were used in the main manuscript.

# 2. Additional Data for Co-In-$ZrO_2$ System

## 2.1. Structural Characterization

**Table S1:** Textural and structural properties of Co-In-$ZrO_2$, Co-$ZrO_2$ and In-$ZrO_2$.

| Catalysts | Co[a)] (atom%) | In[a)] (atom%) | Zr[a)] (atom%) | Crystallite size[b)] (nm) | Surface area[c)] ($m^2\ g^{-1}$) |
|---|---|---|---|---|---|
| Co-In-$ZrO_2$ | 11 (10) | 11 (5) | 78 (85) | 12.9 | 69 |
| Co-$ZrO_2$ | 11 (10) | - | 89 (90) | 10.9 | 65 |
| In-$ZrO_2$ | - | 12 (5) | 88 (95) | 12.2 | 73 |

a) Surface concentration determined by XPS analysis. Values in parenthesis denote theoretical loading b) Average crystallite size calculated from P-XRD data using the Scherrer equation. c) Derived from BET theory.

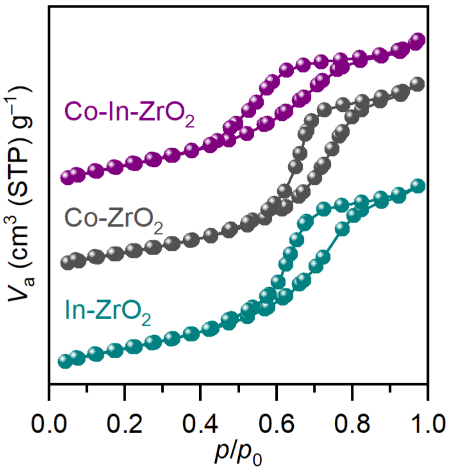


**Figure S1:** $N_2$ adsorption-desorption isotherms of Co-In-$ZrO_2$, Co-$ZrO_2$ and In-$ZrO_2$.

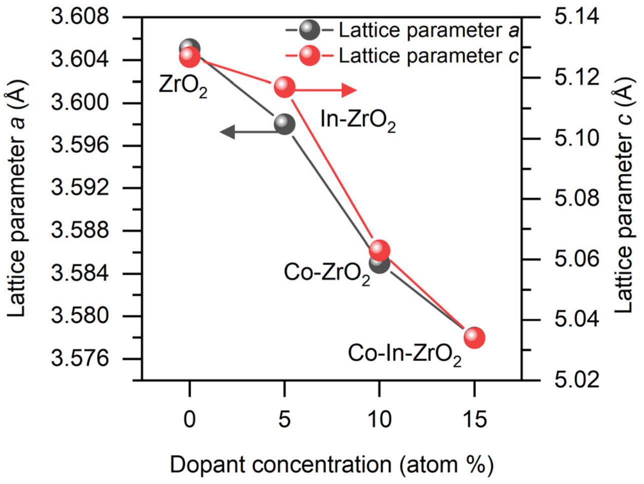


**Figure S2:** Change of lattice parameters of *t*-$ZrO_2$ crystal with respect to change in dopant concentrations in Co-In-$ZrO_2$, Co-$ZrO_2$, In-$ZrO_2$ and $ZrO_2$.

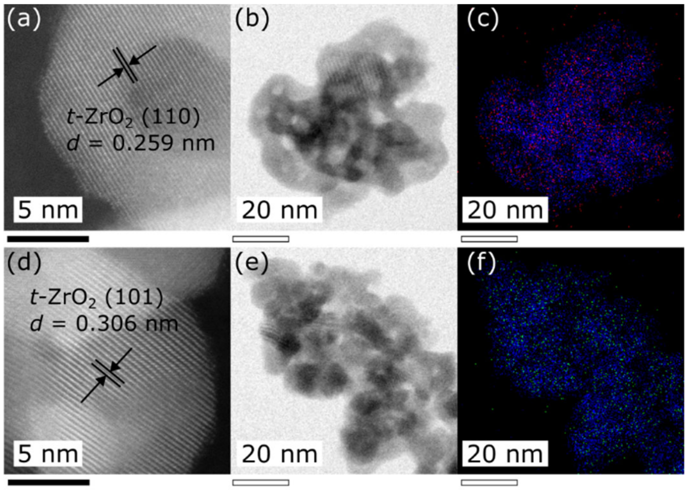


**Figure S3:** (a) STEM analysis of Co-$ZrO_2$. (b, c) Elemental mapping of Co (red) and Zr (blue) in the selected region of the Co-$ZrO_2$ catalyst. (d) STEM analysis of In-$ZrO_2$. (e, f) Elemental mapping of In (green) and Zr (blue) in the selected region of the In-$ZrO_2$ catalyst.

## 2.2. Catalytic Performance

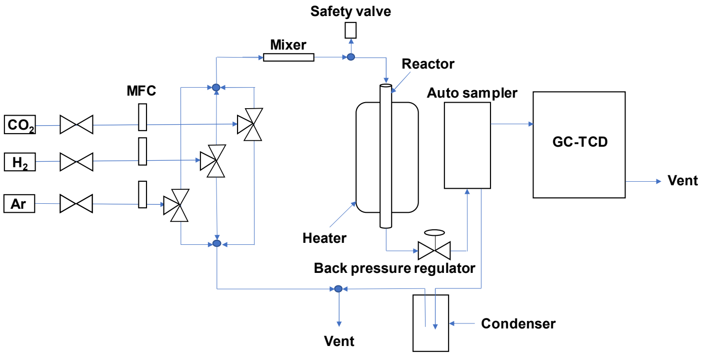


**Figure S4:** Scheme of fixed bed flow reactor setup for catalytic evaluation.

**Table S2:** Overview of catalytic performance of Co-In-$ZrO_2$, Co-$ZrO_2$, In-$ZrO_2$, and $ZrO_2$. Reaction conditions: $p$ = 3 MPa, $GHSV$ = 30000 mL $h^{-1}$ $g_{cat}^{-1}$, $H_2/CO_2$ = 4:1.

| Catalyst | $T$ (°C) | $X_{CO_2}$ (%) | $S_{CO}$ (%) | $S_{MeOH}$ (%) | $STY_{CO}$ (µmol$_{CO}$ $g_{cat}^{-1}$ $s^{-1}$) | $STY_{MeOH}$ (µmol$_{MeOH}$ $g_{cat}^{-1}$ $s^{-1}$) |
|---|---|---|---|---|---|---|
| Co-In-$ZrO_2$ | 300 | 2.7 | 35 | 65 | 0.7 | 1.3 |
| | 270 | 1.0 | 14 | 86 | 0.10 | 0.63 |
| In-$ZrO_2$ | 300 | 2.3 | 56 | 44 | 0.97 | 0.75 |
| | 270 | 0.6 | 42 | 58 | 0.19 | 0.27 |
| Co-$ZrO_2$ | 300 | 2.3 | 71 | 29 | 1.28 | 0.35 |
| | 270 | 0.5 | 61 | 39 | 0.20 | 0.13 |
| Co-$ZrO_2$[a)] + In-$ZrO_2$ | 300 | 2.6 | 62 | 38 | 1.2 | 0.69 |
| $ZrO_2$ | 300 | 0.3 | 100 | 0[b)] | 0.20 | 0[b)] |

a) Physical mixture of Co-$ZrO_2$ and In-$ZrO_2$. b) below detection limit.

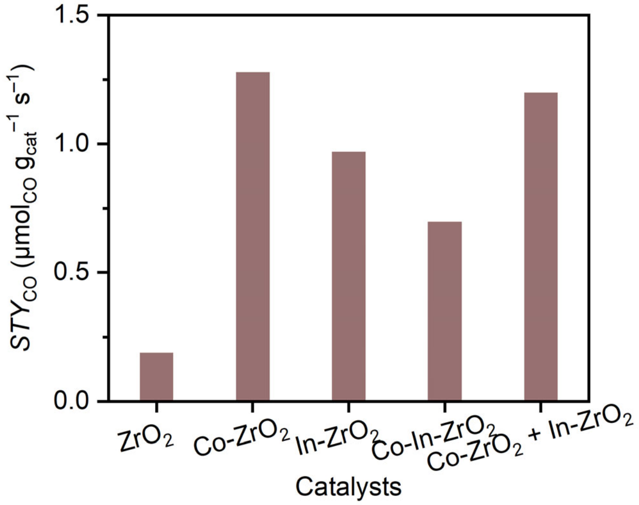


**Figure S5:** Space-time yield of CO over Co-In-$ZrO_2$, the binary counterparts Co-$ZrO_2$ and In-$ZrO_2$, a physical mixture of Co-$ZrO_2$ and In-$ZrO_2$, and undoped $ZrO_2$. Reaction conditions: 300 °C, 3 MPa, 30,000 mL $h^{-1}$ $g_{cat}{}^{-1}$, $H_2/CO_2$ = 4:1.

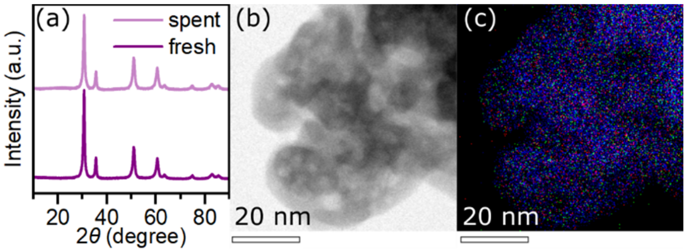


**Figure S6:** (a) P-XRD analysis of fresh and spent Co-In-$ZrO_2$ catalyst. (b, c) Elemental mapping of Co (red), In (green) and Zr (blue) in the spent Co-In-$ZrO_2$ catalyst.

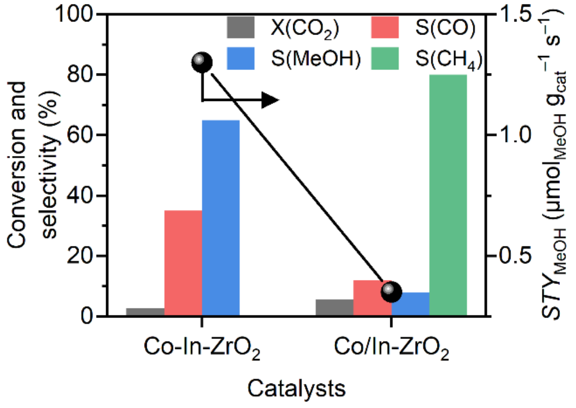


**Figure S7:** Comparison of catalytic activity between Co-In-$ZrO_2$ and Co/In-$ZrO_2$. Reaction conditions: 300 °C, 3 MPa, 30,000 mL $h^{-1}$ $g_{cat}^{-1}$, $H_2/CO_2$ =4. Co/In-$ZrO_2$ catalyst was reduced at 400 °C under $H_2$ for 1 h prior to the reaction.

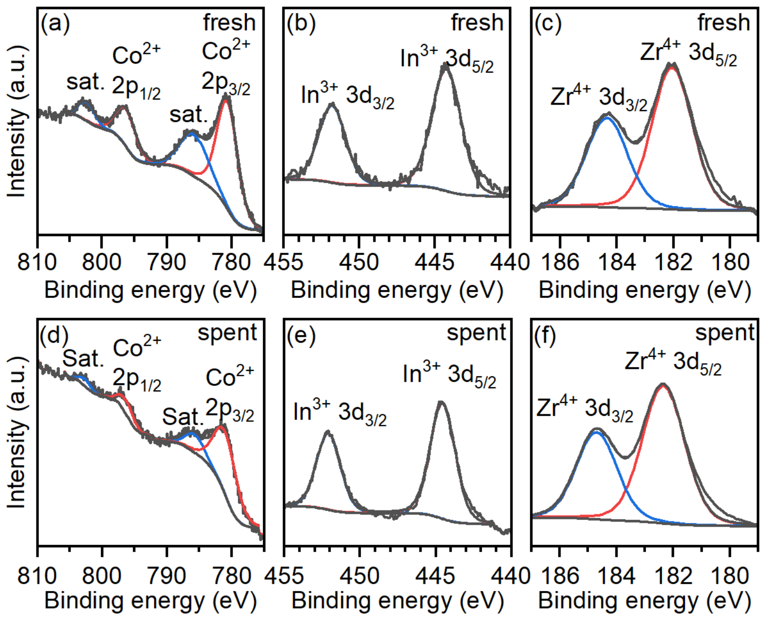


**Figure S8:** (a-c) XPS spectra of Co 2p, In 3d and Zr 3d region of fresh Co-In-$ZrO_2$ catalyst respectively, (d-f) XPS spectra of Co 2p, In 3d and Zr 3d region of used Co-In-$ZrO_2$ catalyst respectively showing the presence of $Co^{2+}$, $In^{3+}$ and $Zr^{4+}$ in both fresh and spent catalysts.

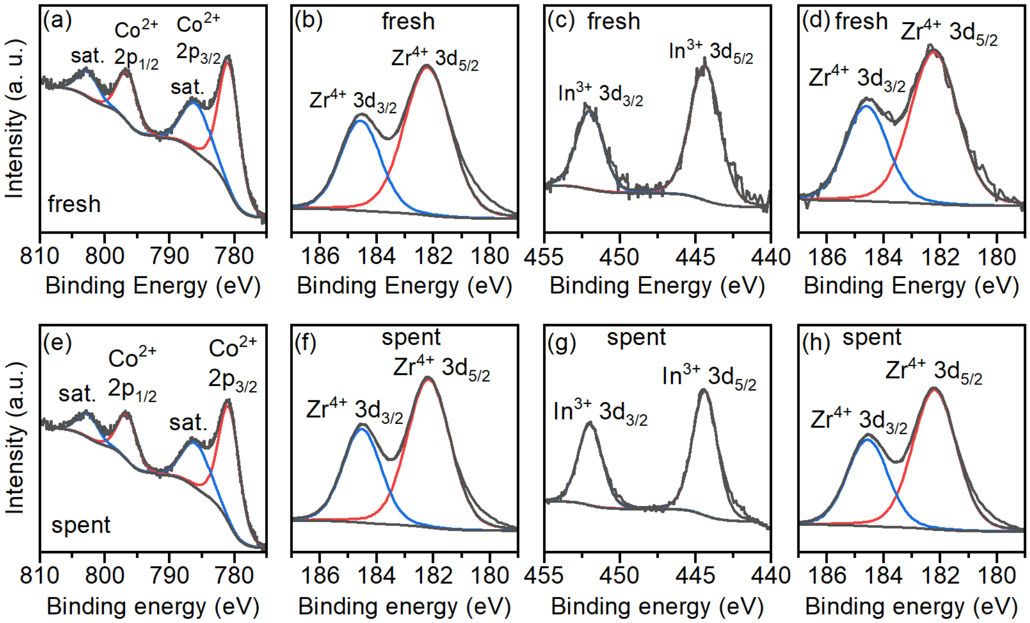


**Figure S9:** XPS spectra of (a, b) fresh and (e, f) spent Co-$ZrO_2$ catalyst. XPS spectra of (c, d) fresh and (g, h) spent In-$ZrO_2$ catalyst.

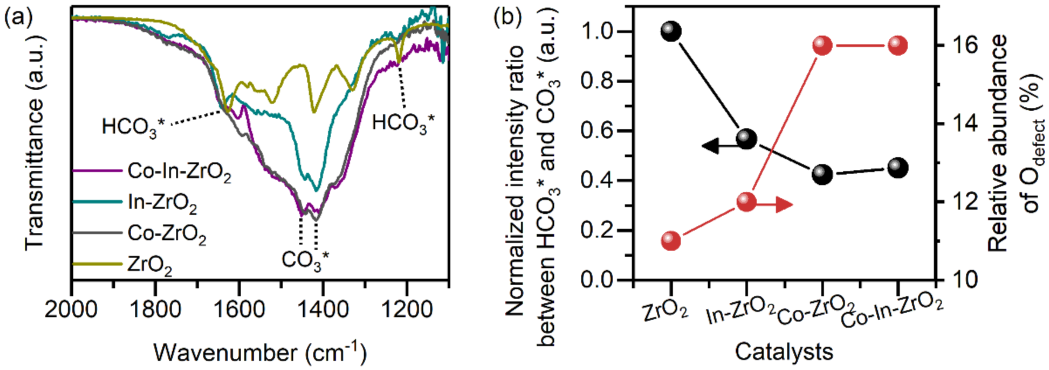


**Figure S10:** (a) IR spectra of adsorbed $CO_2$ showing $HCO_3$* and $CO_3$*. All the spectra were normalized according to the $HCO_3$* peak intensity (1630 $cm^{-1}$). (b) Opposite trend between surface -OH abundance and $O_{defect}$ shown in O 1S XPS analysis over all catalysts.

To understand the surface -OH and oxygen vacancy abundance, we analyzed IR spectra of $CO_2$ adsorption over different catalysts. $CO_2$ in presence of surface -OH groups forms bicarbonate ($HCO_3$*) species and interacts with crystal oxygen on the surface to form carbonate ($CO_3$*) species. This carbonate formation is enhanced when oxygen vacancy is present on the surface. $CO_2$ interacts strongly with the metal atoms (having unsaturated coordination environment) and one crystal oxygen near the vacancy and forms stable $CO_3$* species. Therefore, by comparing the relative intensities of bicarbonate and carbonate, we can qualitatively understand the abundance of -OH and oxygen vacancy on the surface the catalysts.

Figure S10a shows IR spectra of adsorbed $CO_2$. The intensity of all spectra was normalized according to the intensity of $HCO_3$* species (1630 $cm^{-1}$) of pure $ZrO_2$. In the case of pure $ZrO_2$ the $HCO_3$* and $CO_3$* showed similar intensity. In the case of In-$ZrO_2$, $CO_3$* intensity increased. The intensity of $CO_3$* further increased for Co-$ZrO_2$ and Co-In-$ZrO_2$. Therefore, upon doping in $ZrO_2$ crystal, relative abundance of surface oxygen defects increased rather than increasing -OH species. Interestingly, the carbonate peak intensity was similar for both Co-$ZrO_2$ and Co-In-$ZrO_2$. This is in agreement with O1s XPS analysis, which suggested the similar abundance of $O_{defect}$ for Co-$ZrO_2$ and Co-In-$ZrO_2$. Therefore, we can conclude that the $O_{defect}$ peak shown in O1s XPS spectra (figure 3b in main text) has major contribution from defective oxygen species.

Moreover, if we plot normalized intensity ratio between $HCO_3$* and $CO_3$* (as a qualitative measure of -OH and defective oxygen species), we can see that upon doping in $ZrO_2$, relative abundance of -OH species decreased (Figure S10b). It decreased the most when Co was present. This trend was completely opposite to that of $O_{defect}$ abundance suggested by O 1s XPS. Therefore, based on these experiments, we conclude that increase in the abundance of $O_{defect}$ shown in O1s XPS in doped catalysts was due to the increase in oxygen vacancy and not because of -OH species present on the surface.

## 2.3. In situ DRIFTS

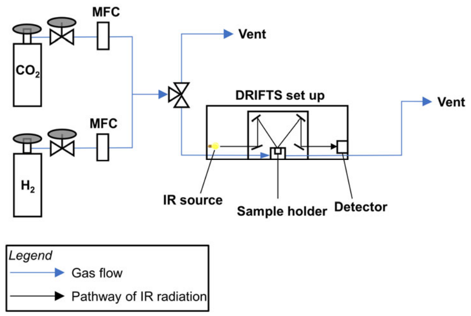


**Figure S11:** Schematic representation of in situ DRIFTS set up.

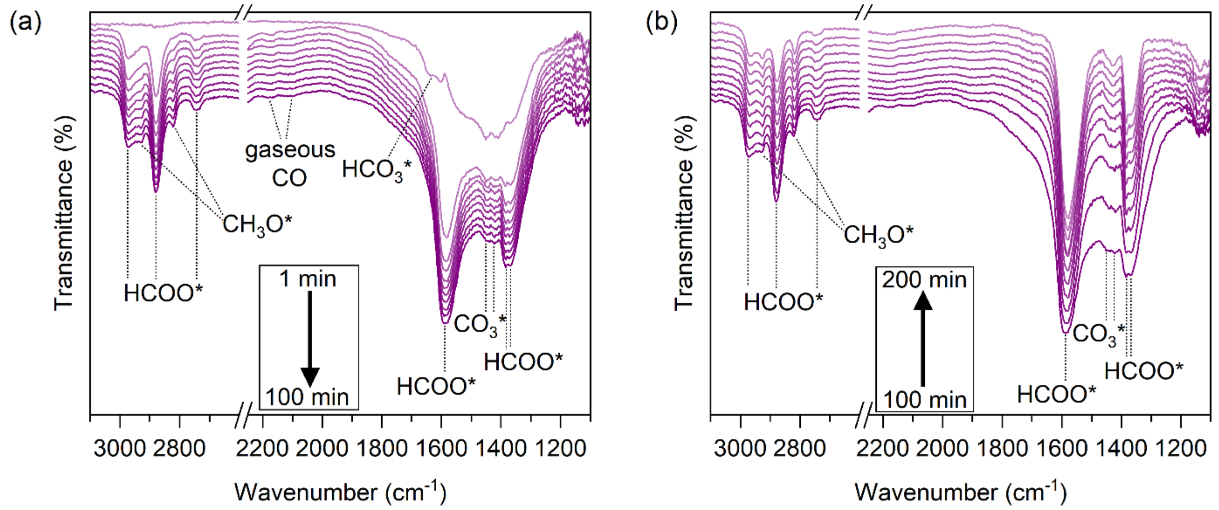


**Figure S12:** In situ DRIFTS study over Co-In-$ZrO_2$. (a) Spectra of first 100 min under $H_2$ + $CO_2$ environment and (b) spectra of following 100 min under $H_2$ atmosphere in the same experiment. Reaction conditions: 300 °C, 0.1 MPa, $H_2/CO_2$ = 4:1 for first 100 min and only $H_2$ for the following 100 min.

At first, we checked whether methanol is formed via the formate pathway. Figure S11 shows the in situ DRIFTS analysis over Co-In-$ZrO_2$. Peak assignments are listed in table S3. At the initial moment of the experiment, when $CO_2$ and $H_2$ were allowed to flow over the catalyst, only carbonate and bicarbonate were observed due to $CO_2$ adsorption (Figure S12a). Soon after, formate emerged as the most abundant species. Methoxy species first appeared after 10 min of reaction and its abundance increased with the continuation of the in situ reaction. After continuing the reaction for 100 min, $CO_2$ flow was stopped. Consequently, first, carbonate species decreased rapidly followed by formate and methoxy (Figure S12b). The same behavior was observed for In-$ZrO_2$. This result indicates that formate species is not a spectator and $CO_2$ hydrogenation to methanol reaction follows the pathway of carbonate to formate to methoxy. Moreover, in situ DRIFTS analysis under CO + $H_2$ environment over Co-In-$ZrO_2$ showed no methoxy formation (Figure S15) confirming methanol formation via formate intermediate.

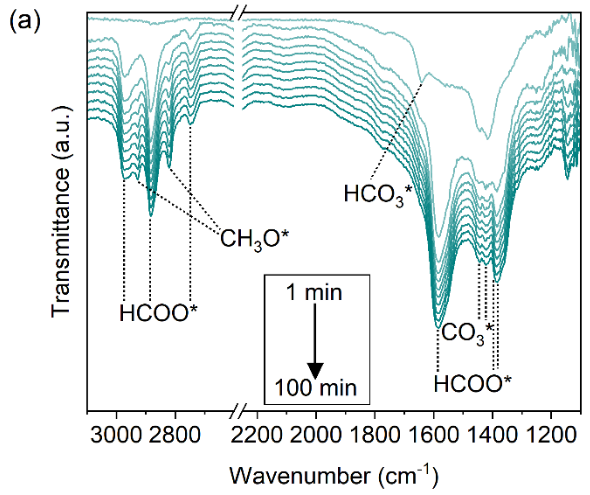


**Figure S13:** In situ DRIFTS study over In-$ZrO_2$. (a) Spectra of first 100 min under $H_2$ + $CO_2$ environment and (b) spectra of following 100 min under $H_2$ atmosphere in the same experiment. Reaction conditions: 300 °C, 0.1 MPa, $H_2/CO_2$ = 4:1 for first 100 min and only $H_2$ for the following 100 min.

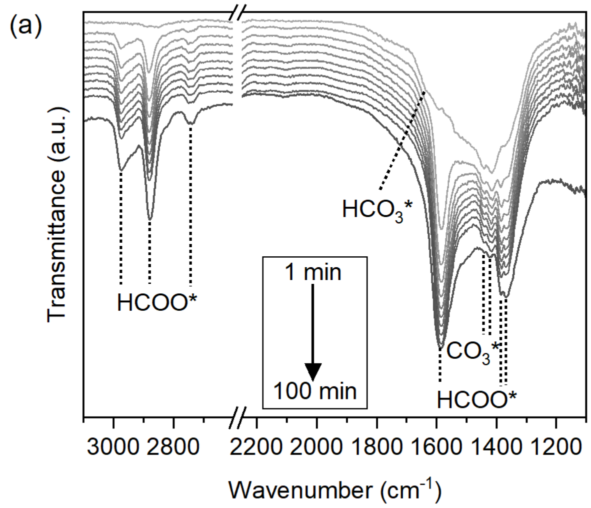


**Figure S14:** In situ DRIFTS study over Co-$ZrO_2$. (a) Spectra of first 100 min under $H_2$ + $CO_2$ environment and (b) spectra of following 100 min under $H_2$ atmosphere in the same experiment. Reaction conditions: 300 °C, 0.1 MPa, $H_2/CO_2$ = 4:1 for first 100 min and only $H_2$ for the following 100 min.

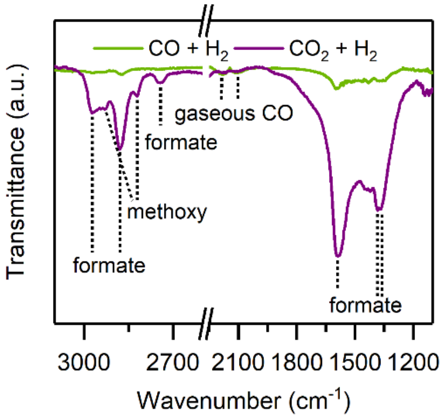


**Figure S15:** Comparison of in situ DRIFTS analysis over Co-In-$ZrO_2$ under $CO_2$ + $H_2$ and CO + $H_2$ environment. Reaction conditions: 300 °C, 0.1 MPa, $H_2/CO_2$ (or CO) = 4:1.

**Table S3:** Assignment of DRIFTS peaks.

| Wavenumber ($cm^{-1}$) | Assignment | Surface Species |
|---|---|---|
| 1587 | $\nu_{as}$(OCO) | Formate ($HCOO^*$) |
| 1367 | $\nu_s$(OCO) | |
| 2878 | $\nu$(CH) | |
| 1384 | $\delta$(CH) | |
| 2968 | $\delta$(CH) + $\nu_{as}$(OCO) | |
| 2742 | $\delta$(CH) + $\nu_s$(OCO) | |
| 1635 | $\nu$(OCOOH) | Bicarbonate ($HCO_3^*$) |
| 1222 | $\delta$(O-H) | |
| 1451, 1415 | $\nu(CO_3)$ | Carbonate ($CO_3^*$) |
| 2926, 2818, 1140 | $\nu(CH_3)$, $\delta$(O-H) | Methoxy ($H_3CO^*$) |

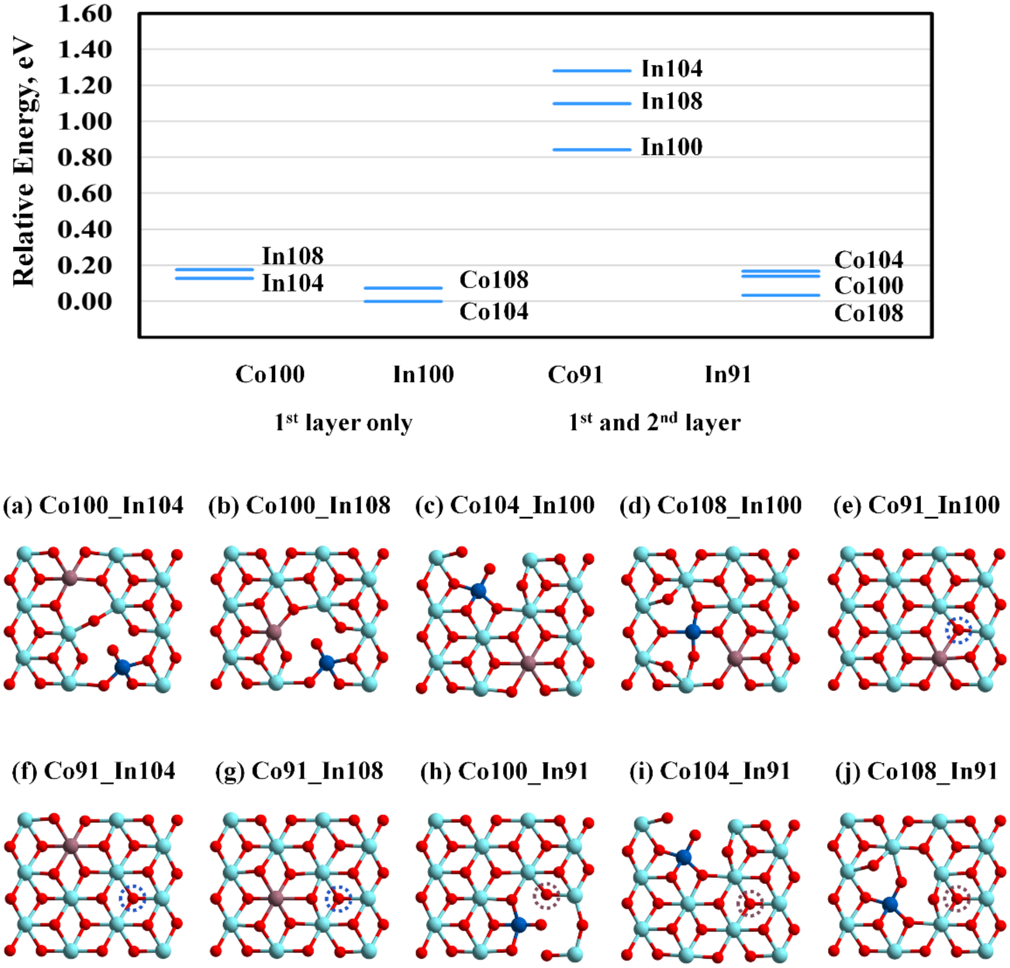


**Figure S16:** The relative energy and the top view of the optimized geometries for In and Co doped $ZrO_2$(101) surface, only the top layer is shown. (a) ~ (d) both Co and In atoms are at the same layer (1$^{st}$ layer), (e) ~ (g) Co atom at the 1$^{st}$ layer and In at the 2$^{nd}$ layer which is indicated by dashed blue cycle. (h) ~ (g) In atom at the 1$^{st}$ layer and Co at the 2$^{nd}$ layer which is indicated by dashed purple cycle. The Zr, O, Co, and In atoms are represented in cyan, red, blue, and purple colors, respectively.

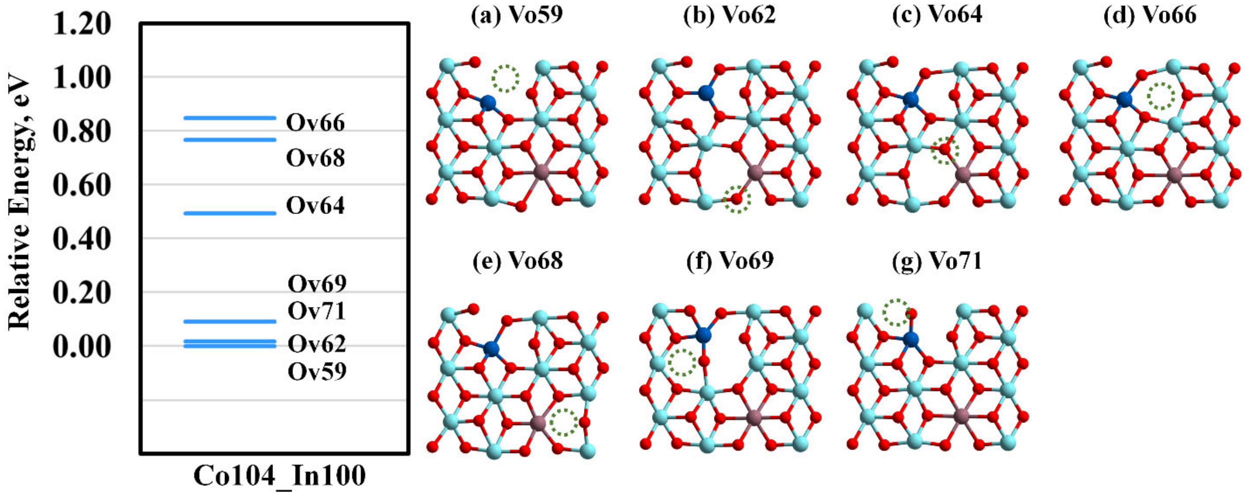


**Figure S17.** The geometries and relative energies for oxygen vacancy on Co-In-$ZrO_2$ surface. The green circle indicates the original position of oxygen atom taken from the most stable geometry, i.e. figure 6a.

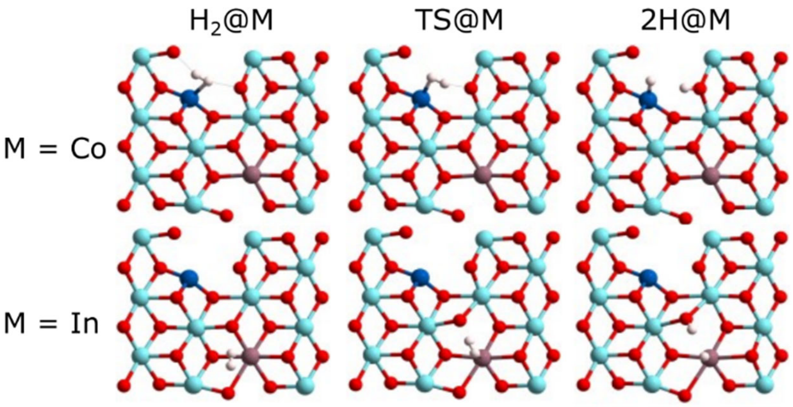


**Figure S18.** The geometries during $H_2$ activation study over clean Co-In-$ZrO_2$ surface. Corresponding relative energies were shown in figure 6e.

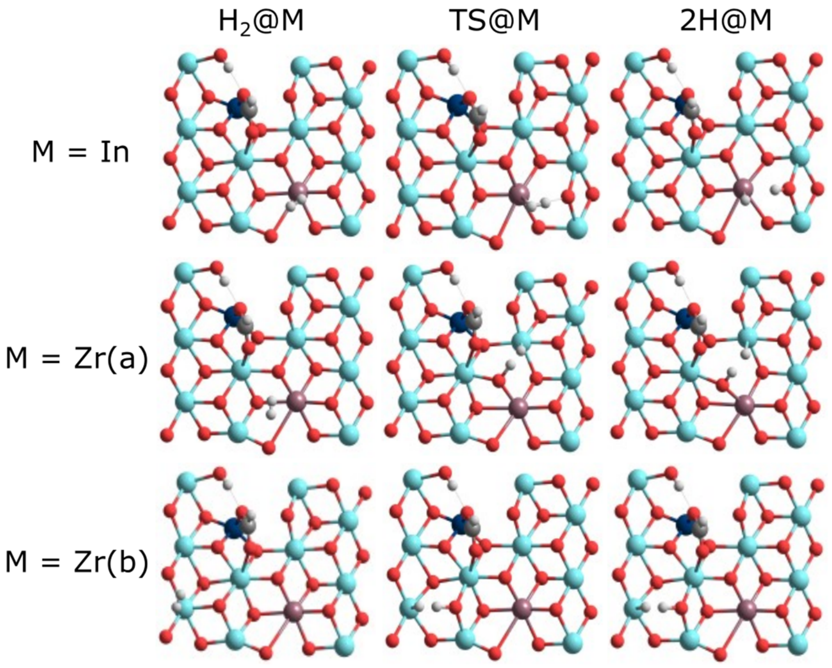


**Figure S19.** The geometries during $H_2$ activation study over formate adsorbed Co-In-$ZrO_2$ surface. Corresponding relative energies were shown in figure 6f.

# 3. Supporting Discussion of Co-Zn-$ZrO_2$ and Co-Ga-$ZrO_2$ Systems

## 3.1. Structural Characterization

Analogously to the Co-In-$ZrO_2$ system, Co-10-Zn-5-$ZrO_2$ (Co-Zn-$ZrO_2$) and Co-10-Ga-5-$ZrO_2$ (Co-Ga-$ZrO_2$) as well as their binary counterparts Zn-5-$ZrO_2$ (Zn-$ZrO_2$) and Ga-5-$ZrO_2$ (Ga-$ZrO_2$) were synthesized and characterized.

Co-Zn-$ZrO_2$, Zn-$ZrO_2$, and Co-Ga-$ZrO_2$ each exhibited a similar surface area in the range of 101 to 112 $m^2\ g^{-1}$. Ga-$ZrO_2$ had a slightly higher surface area of 133 $m^2\ g^{-1}$ (Table S4, figure S20). XRD analysis revealed that all catalysts show peaks of *t*-$ZrO_2$. Phase purity was confirmed by the absence of peaks from individual oxides (Figure S21). The crystallite sizes of the ternary oxides were slightly higher than those of the binary oxides (Table S4). With increasing dopant concentration, the peaks for the (101) plane of *t*-$ZrO_2$ shifted towards higher angles in $2\theta$, indicating the successful incorporation of dopants into the matrix (Figure S22). This phenomenon is explained by the smaller ionic radii of the dopants ($r_{Co^{2+}}$= 0.74 Å, $r_{Zn^{2+}}$ = 0.74 Å, $r_{Ga^{3+}}$ = 0.62 Å) in comparison to zirconium(IV) ($r_{Zr^{4+}}$ = 0.84 Å) and consequent reduction of interplanar spacing.[7]

The catalysts were recovered after catalytic testing and analyzed regarding a potential structural change induced under reaction conditions. The XRD patterns of fresh and spent catalysts (Figure S23-S24) evidence that the reactive environment does not lead to a phase change.

## 3.2. Catalytic Performance

As stated in the main text, the incorporation of Co atoms into Zn-$ZrO_2$ and Ga-$ZrO_2$ leads to an increase of both methanol selectivity and space-time yield just as in the case of the Co-In-$ZrO_2$ system (Table S5). Out of all tested catalysts, Co-Zn-$ZrO_2$ exhibits the highest methanol selectivity and space-time yield. The $CO_2$ conversion over Co-Zn-$ZrO_2$ was similar to Zn-$ZrO_2$. In the case of Co-Ga-$ZrO_2$ the $CO_2$ conversion was higher than over Ga-$ZrO_2$ (Figure S25a). In both Co-Zn-$ZrO_2$ and Co-Ga-$ZrO_2$ the CO production was reduced in comparison to the single atom analogues (Figure S25b).

## 3.3. Investigation of Reaction Mechanism

**$CO_2$ Adsorption and Formate Stabilization Site**

All catalysts were subjected to analysis via $CO_2$-TPD. For all samples, two desorption features were observed (Figure S26). In accordance with the Co-In-$ZrO_2$ system, both the ternary oxides exhibit a higher $CO_2$ adsorption than the binary oxides (Figure S27).

In-situ DRIFTS analysis was carried out over all catalysts under same reaction conditions used for Co-In-$ZrO_2$ (Figure S28-S31). For all catalysts, the common surface intermediate is formate, and its generation was facile, as equilibrium surface coverage is achieved within the first 60 minutes of reaction over all catalysts. Over ternary oxides, methoxy formation and its further hydrogenation to produce methanol was fast as compared to binary oxides. Over Co-Ga-$ZrO_2$ and Ga-$ZrO_2$ we were also able to identify Ga hydride, in line with previous reports on Ga-$ZrO_2$.[8]

In the case of Co-Zn-$ZrO_2$ and Co-Ga-$ZrO_2$, the comparison of the formate peak positions over $ZrO_2$, single atom catalysts (Co-$ZrO_2$, Zn-$ZrO_2$ and Ga-$ZrO_2$), and the dual-atom catalysts (Figure S32) could successfully substantiate our hypothesis of formate stabilization over Co-Zr interfacial site. The peak position of formate in the dual atom catalysts matches with the one over Co-$ZrO_2$.

In addition, we conducted a formic acid temperature-programmed decomposition (HCOOH-TPD) over Co-Zn-$ZrO_2$, Co-Ga-$ZrO_2$, and Zn-$ZrO_2$, Ga-$ZrO_2$ (Figure S33). The observations were in line with the results of Co-In-$ZrO_2$. The HCOOH-TPD study suggests that Co-Zr interfacial sites are mainly responsible for the stabilization of formate in the ternary oxides.

**Cooperation between Co and Zn/Ga atoms for methanol production in ternary oxide**

Analogously to the investigation of the Co-In-$ZrO_2$ system, we utilized the formate and methoxy peak intensities during the in-situ DRIFTS experiments to investigate the synergetic effect of Co and Zn/Ga sites during $CO_2$ hydrogenation (Figure S34-S35). Similar to Co-In-$ZrO_2$, the cooperation between Co and Zn/Ga in Co-Zn-$ZrO_2$ and Co-Ga-$ZrO_2$ facilitated formate hydrogenation to methoxy species and methoxy removal step, which were slower over single atom catalyst counterparts. This co-operative effect is more pronounced in Co-Zn-$ZrO_2$. These analyses confirmed that the Co-Zn-$ZrO_2$ and Co-Ga-$ZrO_2$ shows similar separation of active sites as Co-In-$ZrO_2$.

# 4. Supporting Tables and Figures for Co-Zn-$ZrO_2$ and Co-Ga-$ZrO_2$ Systems

## 4.1. Structural Characterization

**Table S4:** Textural and structural properties of Co-Zn-$ZrO_2$, Zn-$ZrO_2$, Co-Ga-$ZrO_2$, and Ga-$ZrO_2$.

| Catalyst | Co[a] (atom%) | *M*[a] (atom%) | Zr[a] (atom%) | Crystallite size[b] (nm) | Surface area[c] ($m^2\ g^{-1}$) |
|---|---|---|---|---|---|
| Co-Zn-$ZrO_2$ | 10 | 13 | 77 | 15.0 | 107 |
| Zn-$ZrO_2$ | - | 14 | 86 | 9.3 | 101 |
| Co-Ga-$ZrO_2$ | 11 | 10 | 79 | 13.7 | 112 |
| Ga-$ZrO_2$ | - | 12 | 88 | 9.5 | 133 |
| $ZrO_2$ | - | - | 100 | 7.6 | 110 |

a) Surface concentration determined via quantitative XPS analysis. b) Average crystallite size calculated from P-XRD data using the Scherrer equation. c) Derived from BET theory.

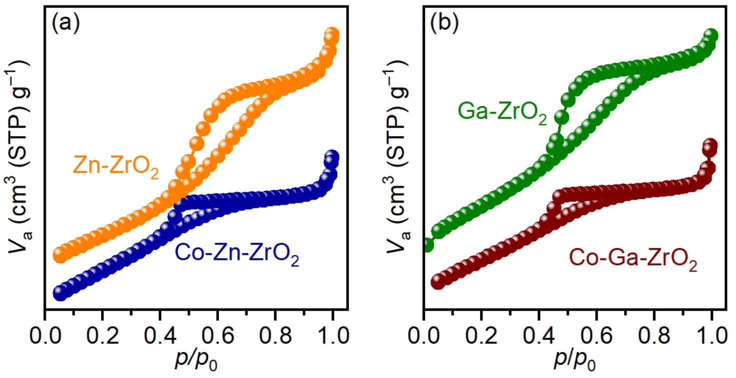


**Figure S20:** $N_2$ adsorption-desorption isotherms for (a) Co-Zn-$ZrO_2$, Zn-$ZrO_2$, and (b) Co-Ga-$ZrO_2$, Ga-$ZrO_2$.

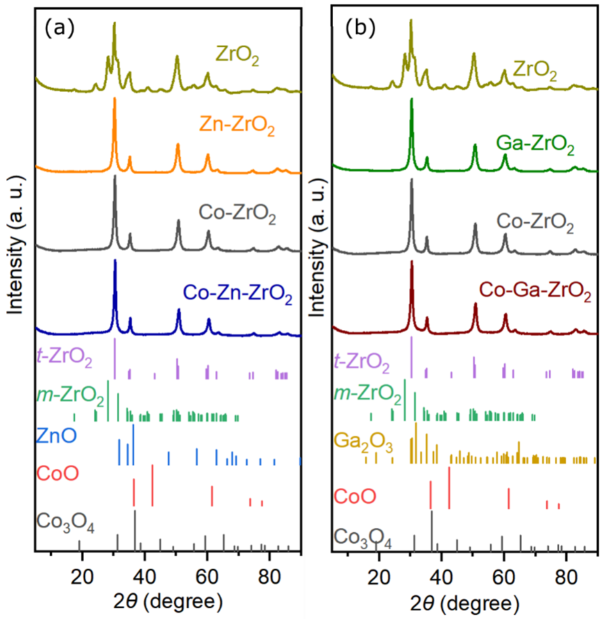


**Figure S21:** P-XRD patterns of Co-In-$ZrO_2$, Co-$ZrO_2$, In-$ZrO_2$, and $ZrO_2$. Reference patterns of phase-pure related oxides (*t*-$ZrO_2$: PDF 00-050-1089; *m*-$ZrO_2$: PDF 00-036-0420; ZnO: PDF 00-036-1451; $Ga_2O_3$: PDF 00-041-1103; CoO: PDF 01-071-1178; $Co_3O_4$: PDF 01-080-1541) are shown in the bottom panel.

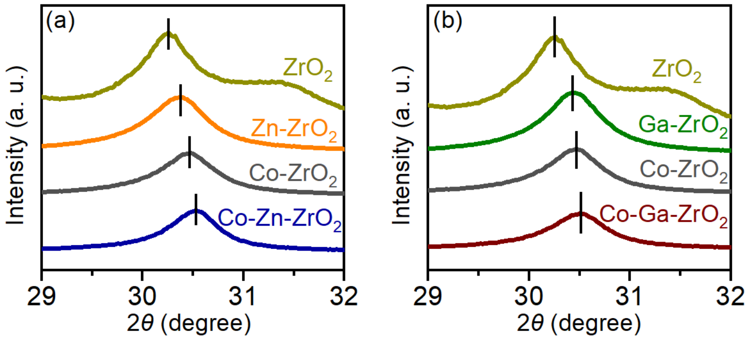


**Figure S22:** Enlargement of the $2\theta$ region from 29° to 32° illustrating the shift of the (101) reflex of *t*-$ZrO_2$ to higher values with increasing dopant concentration.

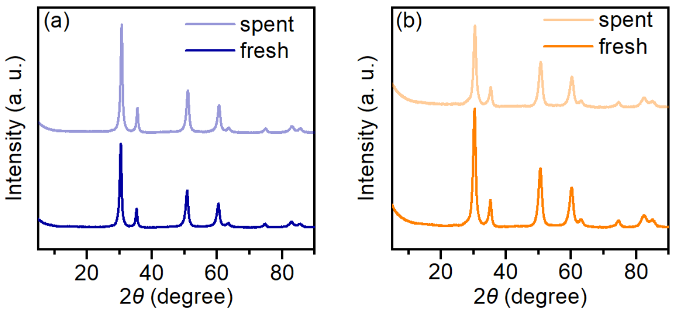


**Figure S23:** Comparison of P-XRD patterns of fresh and spent (a) Co-Zn-$ZrO_2$ and (b) Zn-$ZrO_2$.

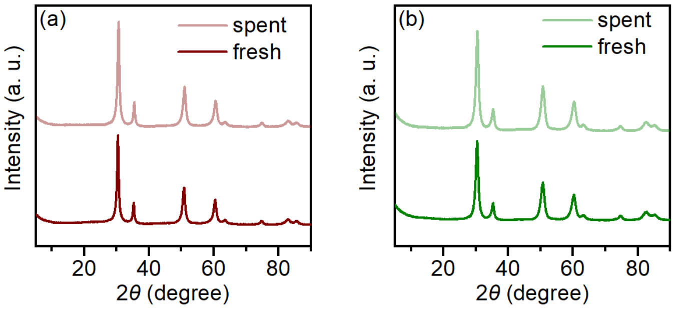


**Figure S24:** Comparison of P-XRD patterns of fresh and spent (a) Co-Ga-$ZrO_2$ and (b) Ga-$ZrO_2$.

## 4.2. Catalytic Performance

**Table S5:** Overview of catalytic performance of Co-Zn-$ZrO_2$, Zn-$ZrO_2$, Co-Ga-$ZrO_2$, Ga-$ZrO_2$, Co-$ZrO_2$, and $ZrO_2$. Reaction conditions: $T$ = 300 °C $p$ = 3 MPa, $GHSV$ = 30,000 mL $h^{-1}$ $g_{cat}^{-1}$, $H_2/CO_2$ = 4:1.

| Catalyst | $X_{CO_2}$ (%) | $S_{CO}$ (%) | $S_{MeOH}$ (%) | $STY_{CO}$ (µmol$_{CO}$ $g_{cat}^{-1}$ $s^{-1}$) | $STY_{MeOH}$ (µmol$_{MeOH}$ $g_{cat}^{-1}$ $s^{-1}$) |
|---|---|---|---|---|---|
| Co-Zn-$ZrO_2$ | 2.9 | 20 | 80 | 0.44 | 1.72 |
| Zn-$ZrO_2$ | 3.0 | 37 | 63 | 0.85 | 1.39 |
| Co-Ga-$ZrO_2$ | 2.2 | 37 | 63 | 0.62 | 1.04 |
| Ga-$ZrO_2$ | 2.0 | 45 | 55 | 0.66 | 0.80 |
| Co-$ZrO_2$ | 2.3 | 71 | 29 | 1.28 | 0.35 |
| $ZrO_2$ | 0.3 | 100 | -[a)] | 0.20 | -[a)] |

a) below detection limit.

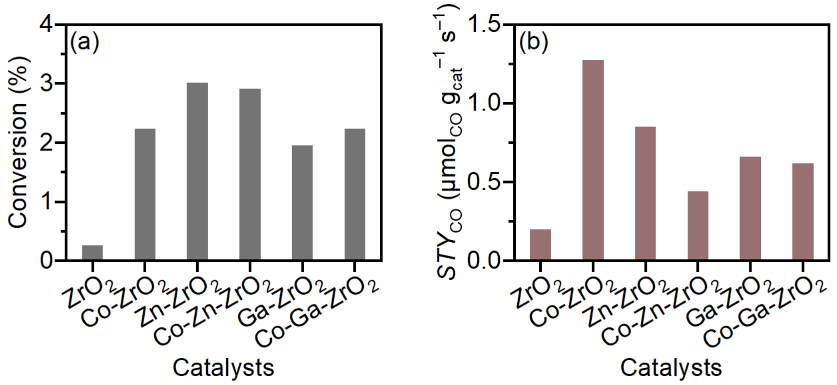


**Figure S25:** (a) $CO_2$ conversion and (b) space time yield of CO over Co-Zn-$ZrO_2$ and Co-Ga-$ZrO_2$ catalysts, their binary counterparts, and undoped $ZrO_2$. Reaction conditions: 300 °C, 3 MPa, 30 000 mL $h^{-1}$ $g_{cat}^{-1}$, $H_2/CO_2$ = 4:1.

## 4.3. Investigation of Reaction Mechanism

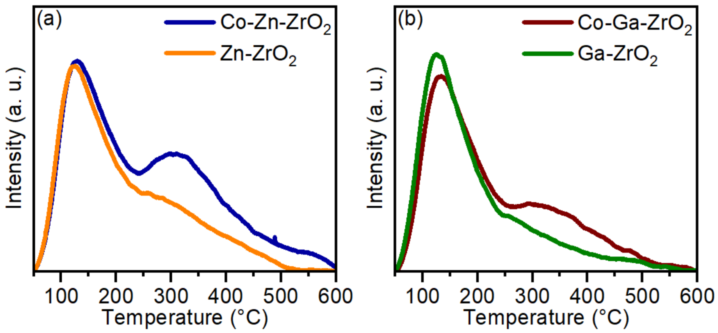


**Figure S26:** $CO_2$ temperature-programmed desorption profiles of (a) Co-Zn-$ZrO_2$, Zn-$ZrO_2$, and (b) Co-Ga-$ZrO_2$, Ga-$ZrO_2$.

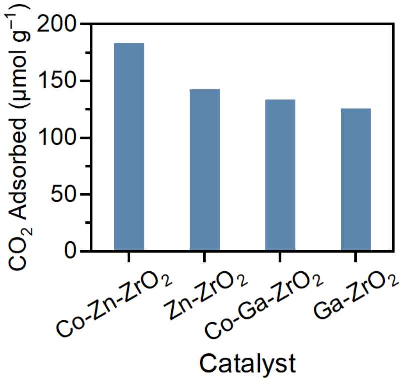


**Figure S27:** Amount of $CO_2$ adsorbed on the surface of Co-Zn-$ZrO_2$, Zn-$ZrO_2$, Co-Ga-$ZrO_2$, and Ga-$ZrO_2$.

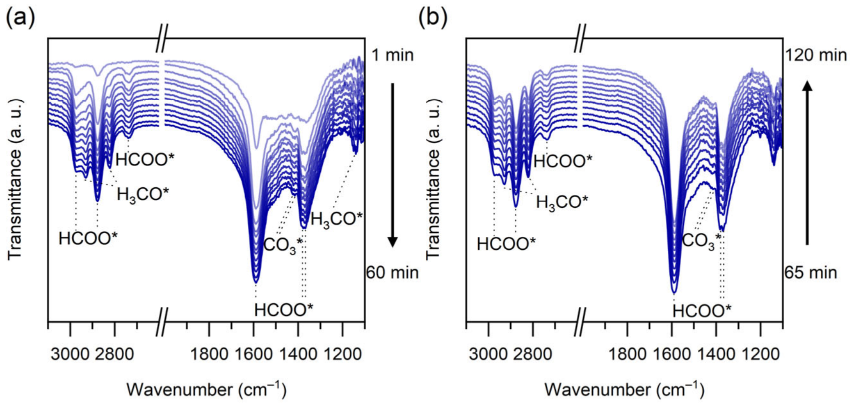


**Figure S28:** In situ DRIFTS study over Co-Zn-$ZrO_2$. (a) Spectra of first 60 min under $H_2$ + $CO_2$ environment and (b) spectra of following 60 min under $H_2$ atmosphere in the same experiment. Reaction conditions: 300 °C, 0.1 MPa, $H_2/CO_2$ = 4:1 for first 60 min and only $H_2$ for the following 60 min.

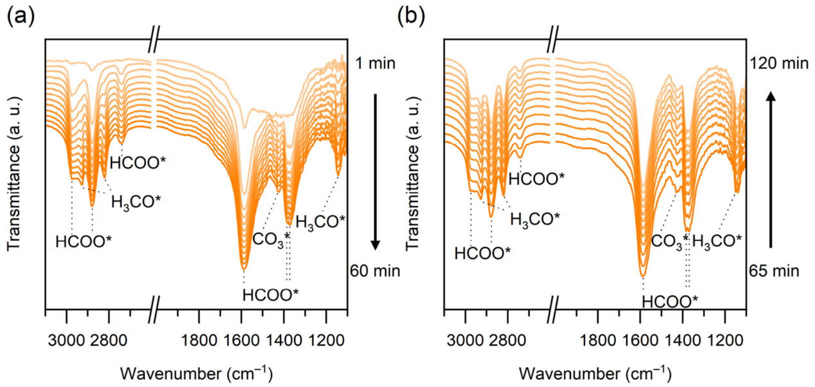


**Figure S29:** In situ DRIFTS study over Zn-$ZrO_2$. (a) Spectra of first 60 min under $H_2$ + $CO_2$ environment and (b) spectra of following 60 min under $H_2$ atmosphere in the same experiment. Reaction conditions: 300 °C, 0.1 MPa, $H_2/CO_2$ = 4:1 for first 60 min and only $H_2$ for the following 60 min.

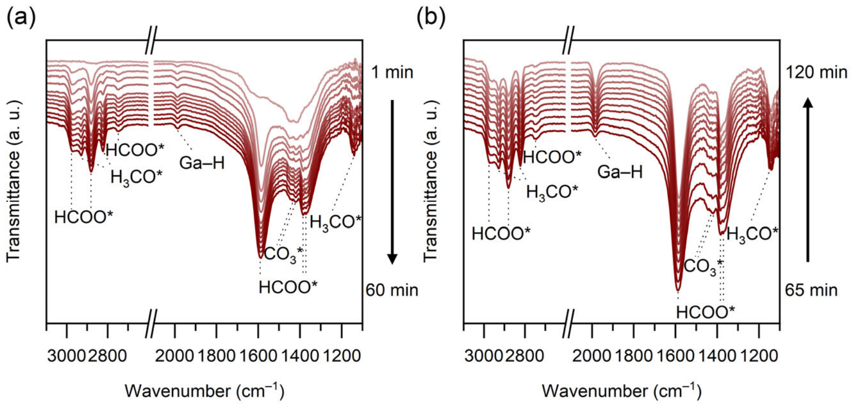


**Figure S30:** In situ DRIFTS study over Co-Ga-$ZrO_2$. (a) Spectra of first 60 min under $H_2$ + $CO_2$ environment and (b) spectra of following 60 min under $H_2$ atmosphere in the same experiment. Reaction conditions: 300 °C, 0.1 MPa, $H_2/CO_2$ = 4:1 for first 60 min and only $H_2$ for the following 60 min.

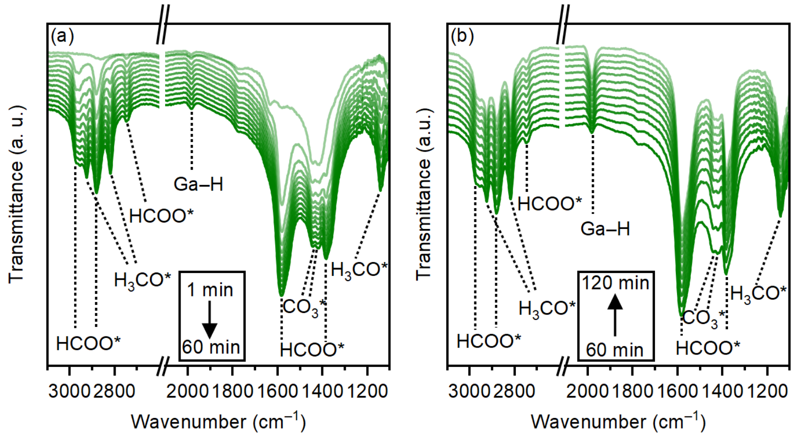


**Figure S31:** In situ DRIFTS study over Ga-$ZrO_2$. (a) Spectra of first 60 min under $H_2$ + $CO_2$ environment and (b) spectra of following 60 min under $H_2$ atmosphere in the same experiment. Reaction conditions: 300 °C, 0.1 MPa, $H_2/CO_2$ = 4:1 for first 60 min and only $H_2$ for the following 60 min.

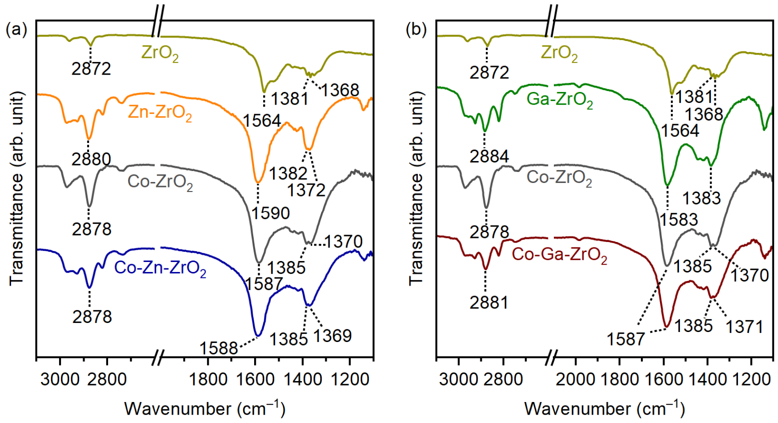


**Figure S32:** Comparison of formate peak positions during in situ DRIFTS experiment for (a) Co-Zn-$ZrO_2$ and (b) Co-Ga-$ZrO_2$ with their binary counterparts and undoped $ZrO_2$. Reaction conditions for DRIFTS experiment: 300 °C, 0.1 MPa, $H_2/CO_2$ = 4:1.

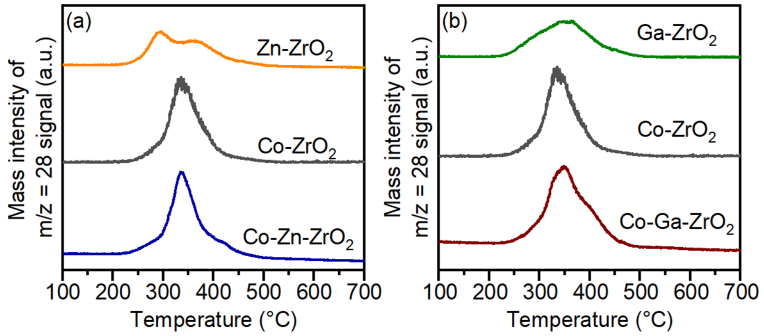


**Figure S33:** Formic acid temperature-programmed decomposition over (a) Co-Zn-$ZrO_2$, Zn-$ZrO_2$ and Co-$ZrO_2$ (b) Co-Ga-$ZrO_2$, Ga-$ZrO_2$ and Co-$ZrO_2$.

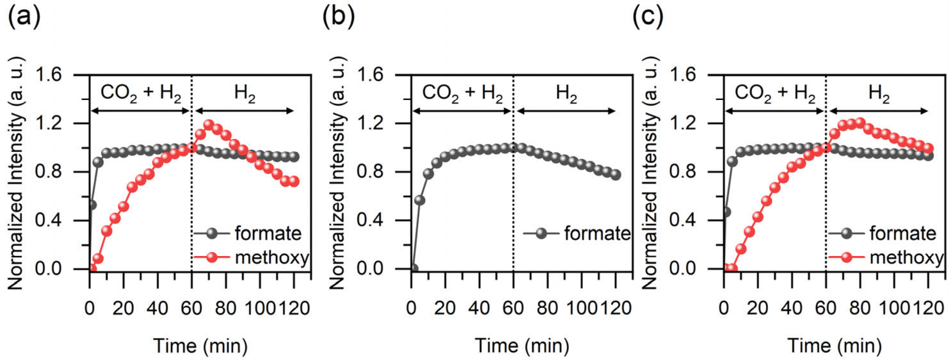


**Figure S34:** Normalized abundance of formate and methoxy species during in situ DRIFTS experiment over (a) Co-Zn-$ZrO_2$, (b) Co-$ZrO_2$, and (c) Zn-$ZrO_2$. For each catalyst, the peak intensity was normalized using the peak intensity of respective species at 60 min during the reaction. Reaction conditions: 300 °C, 0.1 MPa, $H_2/CO_2$ = 4:1.

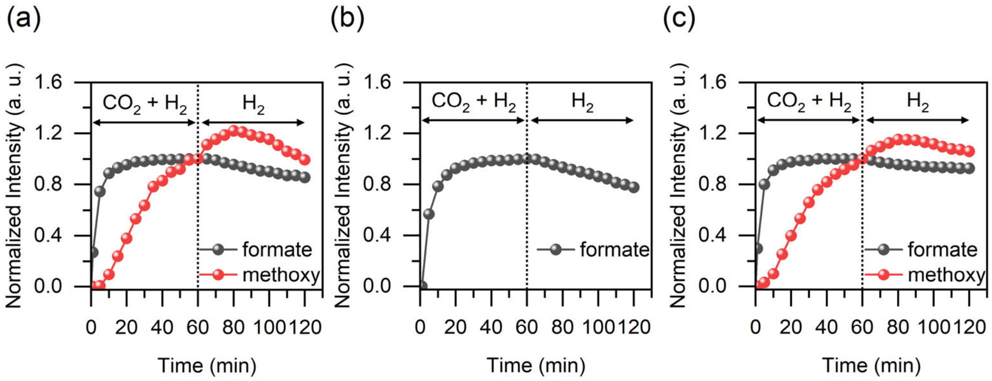


**Figure S35:** Normalized abundance of formate and methoxy species during in situ DRIFTS experiment over (a) Co-Ga-$ZrO_2$, (b) Co-$ZrO_2$, and (c) Ga-$ZrO_2$. For each catalyst, the peak intensity was normalized using the peak intensity of respective species at 60 min during the reaction. Reaction conditions: 300 °C, 0.1 MPa, $H_2/CO_2$ = 4:1.

# 5. Supplementary References

(1) Brunauer, S.; Emmett, P. H.; Teller, E. Adsorption of Gases in Multimolecular Layers. *J. Am. Chem. Soc* **1938**, *60* (2), 309–319.

(2) Kresse, G.; Furthmüller, J. Efficient Iterative Schemes for Ab Initio Total-Energy Calculations Using a Plane-Wave Basis Set. *Phys. Rev. B* **1996**, *54* (16), 11169–11186.

(3) Perdew, J. P.; Burke, K.; Ernzerhof, M. Generalized Gradient Approximation Made Simple. *Phys. Rev. Lett.* **1996**, *77* (18), 3865–3868.

(4) Teufer, G. The Crystal Structure of Tetragonal $ZrO_2$. *Acta Crystallogr.* **1962**, *15* (11), 1187.

(5) Maeda, S.; Harabuchi, Y.; Sumiya, Y.; Takagi, M.; Suzuki, K.; Hatanaka, M.; Osada, Y.; Taketsugu, T.; Morokuma, K.; Ohno, K. "GRRM17" can be found under http://iqce.jp/GRRM/index_e.shtml, **2021** (accessed 22nd November 2023).

(6) Maeda, S.; Ohno, K.; Morokuma, K. Systematic Exploration of the Mechanism of Chemical Reactions: The Global Reaction Route Mapping (GRRM) Strategy Using the ADDF and AFIR Methods. *Phys. Chem. Chem. Phys*. **2013**, *15*, 3683–3701.

(7) Shannon, R. D. Revised Effective Ionic Radii and Systematic Studies of Interatomic Distances in Halides and Chalcogenides. *Acta Crystallogr. Sect. A* **1976**, *32* (5), 751–767.

(8) Feng, W. H.; Yu, M. M.; Wang, L. J.; Miao, Y. T.; Shakouri, M.; Ran, J.; Hu, Y.; Li, Z.; Huang, R.; Lu, Y. L.; Gao, D.; Wu, J. F. Insights into Bimetallic Oxide Synergy during

Carbon Dioxide Hydrogenation to Methanol and Dimethyl Ether over $GaZrO_x$ Oxide Catalysts. *ACS Catal.* **2021**, *11* (8), 4704–4711.
